# Induced Treg‐Derived Extracellular Vesicles Suppress CD4^+^ T‐Cell‐Mediated Inflammation and Ameliorate Bone Loss During Periodontitis Partly Through CD73/Adenosine‐Dependent Immunomodulatory Mechanisms

**DOI:** 10.1002/jev2.70118

**Published:** 2025-07-07

**Authors:** Carolina Rojas, Michelle García, Luis González‐Osuna, Mauricio Campos‐Mora, Enrique Ponce de León, Alfredo Sierra‐Cristancho, Claudia Terraza, Cristian Cortez, Luis Daniel Sansores‐España, Paola Carvajal, Jordan Bazoer, Qi Peng, Charlotte Lawson, Lesley A. Smyth, Karina Pino‐Lagos, Rolando Vernal

**Affiliations:** ^1^ Laboratorio de Biología Periodontal, Faculty of Dentistry Universidad de Chile Santiago Chile; ^2^ Centro de Investigación e Innovación Biomédica, Faculty of Medicine Universidad de Los Andes Santiago Chile; ^3^ Faculty of Dentistry Universidad de los Andes Santiago Chile; ^4^ Departamento de Rehabilitación Oral, Faculty of Dentistry Universidad de Chile Santiago Chile; ^5^ Faculty of Dentistry Universidad Andres Bello Santiago Chile; ^6^ Escuela de Tecnología Médica, Faculty of Sciences Pontificia Universidad Católica de Valparaíso Valparaíso Chile; ^7^ School of Medicine and Biomedical Sciences University of West London London UK; ^8^ Centre for Nephrology, Urology and Transplantation, School of Immunology and Mucosal Biology King's College London London UK; ^9^ Comparative Biomedical Sciences Royal Veterinary College London UK; ^10^ School of Pharmacy and Biomedical Sciences UCLan Preston UK

**Keywords:** 5’‐nucleotidase, adenosine, extracellular vesicles, periodontitis, regulatory T cell, retinoic acid, Treg

## Abstract

Regulatory T cell (Treg)‐derived extracellular vesicles (EVs) represent a contact‐independent mechanism by which Tregs suppress dysregulated immune responses. These EVs carry diverse immunomodulatory molecules, including CD73, an ectoenzyme that hydrolyses AMP into adenosine. Adenosine subsequently acts as a potent immunosuppressive mediator that inhibits effector CD4⁺ T cell activation and controls pathological inflammation. Periodontitis is a highly prevalent inflammatory disease characterised by the accumulation of IL‐17A‐expressing CD4⁺ T cells in response to dysbiotic oral bacterial biofilms, ultimately leading to RANKL‐mediated alveolar bone resorption and tooth loss. We tested the hypothesis that CD73⁺ Treg‐derived EVs, isolated from Tregs induced with polarising cytokines in the presence of retinoic acid, could limit inflammation and prevent alveolar bone loss in periodontitis. Our findings demonstrate that Tregs induced with polarising cytokines in the presence of retinoic acid express high levels of CD73 and secrete adenosine‐producing suppressive CD73^+^ EVs. Furthermore, local administration of these CD73⁺ Treg‐derived EVs in a murine periodontitis model reduced activated CD4⁺ T cell infiltration, decreased IL‐17A and RANKL expression, and attenuated osteoclast‐mediated alveolar bone loss. In conclusion, retinoic acid‐induced Treg‐derived EVs suppress CD4⁺ T cell‐driven inflammation and ameliorate periodontitis, at least in part through CD73/adenosine‐dependent immunomodulatory mechanisms.

## 1 Introduction

Regulatory T cells (Tregs) are critical modulators of immunity that protect the host against deregulated inflammatory responses and autoimmune diseases (Ge et al. 2024). Tregs are phenotypically characterised by the expression of the Forkhead box P3 (Foxp3) transcription factor, which controls their differentiation and phenotypic stability (Ge et al. 2024). In addition, Tregs constitutively express high levels of CD25, the alpha‐chain of the interleukin (IL)‐2 receptor, determining part of their suppressor activities (Bayati et al. 2020). Tregs are naturally generated in the thymus (nTregs) or are induced peripherally (iTregs) from naïve T cells following antigen recognition in the presence of transforming growth factor (TGF)‐β and IL‐2 (Bayati et al. 2020). iTregs can also be generated in vitro from CD4^+^Foxp3^−^ T cells when exposed to polarising cytokines (Kanamori et al. 2016). Other factors, such as the vitamin‐A‐derived metabolite termed *all‐trans* retinoic acid (RA), contribute to iTregs lineage consolidation, phenotypic stability and suppressive function (Kanamori et al. 2016; Pino‐Lagos et al. 2010).

In addition to the cell‐to‐cell contact‐dependent mechanisms, Tregs can efficiently exert immune suppression by releasing extracellular vesicles (EVs) (Rojas et al. 2020). Treg‐derived extracellular vesicles (TEVs) carry multiple intravesicular or on‐surface immunosuppressive factors, such as cytokines, miRNAs and enzymes (Chen et al. 2021; Rojas et al. 2020; Tung et al. 2020). One of these is CD73 (Smyth et al. 2013), an ectoenzyme that extracellularly catalyses the hydrolysis of adenosine monophosphate (AMP) into adenosine (ADO), a nucleoside with strong immunosuppressive capacities (de Oliveira Bravo et al. 2016). ADO is a purinergic nucleoside base that interacts with four G protein‐coupled receptors (GPCRs): A_1_R, A_2A_R, A_2B_R and A_3_R expressed by immune and non‐immune cells and thus exercising their activities in a wide repertoire of cells (de Oliveira Bravo et al. 2016). Indeed, CD73‐generated ADO acts as a potent anti‐inflammatory factor in several inflammatory diseases by interacting with A_2A_R or A_2B_R‐expressing cells (Cheng et al. 2022). Additionally, ADO shows potent suppressive effects over effector T cells, predominantly expressing the high‐affinity receptor A_2A_R (Gourdin et al. 2018). ADO‐A_2A_R interaction inhibits T‐cell receptor (TCR)‐mediated cell signalling by upregulating intracellular cyclic adenosine monophosphate (cAMP) production and protein kinase A (PKA) activation, which consequently suppresses effector T‐cell activation, proliferation and pro‐inflammatory cytokine release, contributing to inflammation restraint (Allard et al. 2012; Gourdin et al. 2018).

Among inflammatory diseases, periodontitis is a highly prevalent disease that causes resorption of teeth‐supporting alveolar bone and, consequently, teeth loss (Hajishengallis and Chavakis 2021). Furthermore, periodontitis favours the development of low‐grade systemic inflammation, which affects the pathogenesis of several other diseases, including type II diabetes mellitus, Alzheimer's disease, cardiovascular disorders, cognitive disorders and adverse pregnancy outcomes (Genco and Sanz, 2020; Hajishengallis and Chavakis 2021). Pathologically, periodontitis is triggered by a deregulated immune response against pathogenic oral bacteria, leading to harmful inflammation around the teeth (Hajishengallis and Chavakis 2021). This disease is characterised by a reduced capacity of Foxp3^+^ Tregs to suppress the activity of pro‐inflammatory Th17 lymphocytes, who are direct producers of the pro‐osteolytic factor termed receptor activator of nuclear factor κB ligand (RANKL) (Alvarez et al. 2019). In addition, Foxp3^+^ Tregs can become phenotypically unstable and acquire a Th17‐like phenotype that produces IL‐17A and RANKL, further contributing to periodontal breakdown (Tsukasaki et al. 2018). In the present study, it was demonstrated that Tregs induced in the presence of retinoic acid (RATregs) are enriched in CD73 and secrete EVs (RATEVs) that contain enzymatically active CD73. These RATEVs promote ADO production following AMP exposure and suppress CD4^+^ T cell activation. In addition, when RATEVs are administered locally in periodontitis‐affected mice, they reduce IL‐17A^+^ and RANKL^+^ CD4^+^ T‐cell infiltration and ameliorate tooth‐supporting alveolar bone loss. These findings reveal the immunosuppressive and bone‐protective capacity of RATEVs in periodontitis and suggest that these effects are mediated, at least in part, through the CD73/adenosine signalling pathway.

## 2 Materials and Methods

### 2.1 Animals

In this study, ∼8‐week‐old male and female mice were used. RATregs were generated from splenic CD4^+^ T lymphocytes isolated from Foxp3^GFP+^ transgenic mice (Strain 023800, The Jackson Laboratory, ME, USA), which express Green Fluorescent Protein (GFP) under the control of the Foxp3 promoter. This mouse strain was chosen because the intensity of GFP fluorescence is directly proportional to the Foxp3 transcript level, enabling the precise assessment of Foxp3^+^ Treg purity by flow cytometry. Experimental periodontitis was induced in C57BL/6 wild‐type mice. Throughout the study, animals were maintained under pathogen‐free conditions at the Institutional Animal Facilities of the Faculty of Dentistry, Universidad de Chile (ODO‐UCH), and the Faculty of Medicine, Universidad de los Andes (FM‐UA). Animals were housed under controlled environmental conditions, including a 12 h light/dark cycle with lights on at 07:00 am, a temperature of 24±0.5°C, relative humidity of 40%–70% and an air exchange rate of 15 room volumes/h. Male and female mice were housed in separate cages with free access to sterile standard food and water, and female mice were confirmed not to be in oestrus during the study. The study was approved by the local Institutional Animal Care and Use Committees (Protocols 18173‐ODO‐UCH, 22578‐ODO‐UCH and CEC2021017 FM‐UA) and conducted under the ARRIVE guidelines. All the experiments were carried out following the American Veterinary Medical Association (AVMA) recommendations and the guidelines approved by the Council of the American Psychological Society (1980) for animal experiments.

### 2.2 RATregs Induction and Characterisation

From male and female C57BL/6 Foxp3^GFP+^ mice‐derived splenic cells (pooled from 1 or 2 mice), total CD4^+^ T cells were magnetically isolated using the EasySep Mouse CD4^+^ T cell isolation kit (Stem Cell Technologies, Vancouver, Canada). Next, isolated CD4^+^ T cells were differentiated into RATregs in EV‐free media. EV‐free media consisted of RPMI media supplemented with Hepes (Gibco, MD, USA), penicillin/streptomycin (Corning, NY, USA), β‐mercaptoethanol (Sigma, MO, USA) and 10% EV‐free foetal bovine serum (FBS) (ThermoFisher, IL, USA), previously subjected to ultracentrifugation at 100,000 × *g* for 16 h followed by filtration using a 0.22 µm filter (Millipore, MA, USA). For RATreg generation, isolated CD4^+^ T cells were cultured at 2 × 10^5^ cells/mL in 24‐well plates coated with anti‐CD3ε (10 µg/mL, clone 145‐2C11, BioXCell, NH, USA) and anti‐CD28 (1 µg/mL, clone 37.51, Biolegend, CA, USA), in the presence of TGF‐β1 (10 ng/mL, ThermoFisher), IL‐2 (100 IU/mL, ThermoFisher) and RA (10 nM, Sigma) for 5 days at 37°C, 5% CO_2_, and 21% O_2_. Additionally, CD4^+^ T cells were differentiated into iTregs using the aforementioned polarising cytokines without RA, and nTregs were isolated from spleens using the CD4^+^CD25^+^ Regulatory T Cell Isolation Kit (Miltenyi Biotec, Bergisch Gladbach, Germany), according to the manufacturer's instructions. The expression of CD4, CD25 and CD73 was analysed in nTregs, iTregs and RATregs by flow cytometry using the following monoclonal antibodies: Anti‐CD4 (clone RM4‐5, Biolegend), anti‐CD25 (clone PC61, Biolegend) and anti‐CD73 (clone TY/11.8, Biolegend). The Foxp3 expression was detected based on the GFP signal. Cell viability was determined using a live/dead kit (LIVE/DEAD Fixable Blue Dead Cell Stain Kit, ThermoFisher). Flow cytometer analysis was performed using a FACSCanto II (BD Biosciences, OR, USA) and data analysed using FlowJo v10 software (Beckton Dickinson, OR, USA), applying a sequential gating strategy based on FSC‐A/SSC‐A parameters, FSC‐A/FSC‐H singlet discrimination and live/dead cell staining.

### 2.3 RATEVs Isolation and Characterisation

RATEVs were isolated from the conditioned media of RATregs using a standardised centrifugation and ultracentrifugation protocol (Théry et al. 2006). Briefly, cells and cell debris were removed from conditioned media by serial centrifugation at 300 × *g* for 5 min, 2000 × *g* for 20 min and two cycles of 10,000 × *g* for 30 min. After filtration, using a 0.22 µm filter (Millipore), RATEVs were isolated and washed with filtered PBS by ultracentrifugation at 100,000 × *g* for 90 min at 4°C, using a Sorvall WX80 ultracentrifuge with a TH‐641 swinging bucket rotor (ThermoFisher). Isolated RATEVs were resuspended in 100 µL of filtered PBS or EV‐free supplemented RPMI media and stored at −80°C until further use. The RATEV concentration and size distribution were determined by nanoparticle tracking analysis (NTA) using a NanoSight NS300 (Malvern Instruments, Malvern, UK), previously calibrated with 100 nm polystyrene latex beads (Malvern Panalytical, Malvern, UK). In addition, RATEVs were analysed using transmission electron microscopy (TEM). For this, RATEV samples were loaded on a formvar‐carbon‐coated grid and placed in UranyLess solution for 1 min, air‐dried for 5 min before being analysed using the TEM microscope Talos F200C G2 (ThermoFisher).

### 2.4 Western Blot Analysis

The presence of CD73 ectoenzyme, CD81 tetraspanin and CD9 on RATEVs was analysed by Western blot following a protocol described elsewhere (Campos‐Mora et al. 2022). Before electrophoresis, protein concentrations were determined using a Pierce BCA Protein Assay kit (ThermoFisher). Immunoblot was performed using the primary antibodies anti‐CD73 (clone 1D7, ThermoFisher), anti‐β‐actin (clone 8H10D10, Cell Signaling Technologies, MA, USA), anti‐CD81 (clone B‐11, Santa Cruz Biotechnologies, CA, USA) and anti‐CD9 (clone C‐4, Santa Cruz Biotechnologies). The goat anti‐mouse IgG (H+L) Alexa Fluor 680 (ThermoFisher) was used as a secondary antibody.

### 2.5 Bead‐Assisted Conventional Flow Cytometry

The presence of CD73 was also analysed by conventional flow cytometry. For this, 2 × 10^9^ RATEVs were adhered to aldehyde/sulphate latex beads (#A37304, Molecular Probes, Sigma) and incubated with the primary antibody anti‐CD73 (clone 1D7, ThermoFisher) for 30 min at room temperature under rotation as previously described (Figueroa‐Valdés et al. 2021). Beads were washed with filtered PBS at 8000 × *g* for 2 min and blocked with 10% BSA for 30 min, under rotation. Finally, beads‐bound RATEVs were incubated with the secondary antibody Alexa Fluor 488 anti‐mouse IgG1 (Biolegend) for 30 min. CD73 expression was assessed by flow cytometry using FACSCanto II (BD Biosciences). The omission of the primary antibody or both antibodies was used as a control to discard any unspecific signal or basal autofluorescence, respectively.

### 2.6 Imaging Flow Cytometry

To confirm the presence of CD73 in RATregs and RATEVs, imaging flow cytometry assays were performed using a Cytek Amnis ImageStreamX Mk II Imaging Flow Cytometer (Cytek Biosciences, CA, USA). RATregs were incubated for 30 min with the monoclonal antibodies anti‐CD73 (clone TY/11.8, Biolegend), anti‐CD4 (clone GK1.5, Biolegend) and anti‐CD25 (clone 3C7, Biolegend). Alternatively, RATEVs obtained from RATregs previously labelled with CFSE following the manufacturer's instructions (ThermoFisher) were incubated for 1 h with the anti‐CD73 monoclonal antibody coupled to the phycoerythrin (PE) fluorophore. RATregs images were acquired at 40× magnification, with low speed and beads removal option deactivated, and 60× magnification, with low speed, maximum laser intensity, central core at 7 µm and beads removal option deactivated, using the IDEAS v.6.2 software as previously described (Tertel et al. 2020).

### 2.7 Phosphate Production

The potential of CD73 to hydrolyse 5’‐AMP and produce inorganic phosphate and ADO was analysed in RATregs and RATEVs. To quantify inorganic phosphate production from 5’‐AMP substrate (Sigma), a Malachite Green colourimetric assay was performed using the SensoLyte kit (AnaSpec, CA, USA). RATregs and RATEVs were washed and suspended in phosphate‐free buffer (PFB). Similarly, the 5’‐AMP substrate and the CD73 inhibitor adenosine 5′‐(α, β‐methylene)‐diphosphate (Merck, Darmstadt, Germany) were prepared in PFB to a final concentration of 50 and 100 µM, respectively. In 96‐well flat‐bottom plates, 2.5, 5, or 10 × 10^4^ RATregs or total RATEVs obtained from 1 or 2 × 10^6^ RATregs were incubated with/without 5’‐AMP substrate for 30 min. To ratify the CD73‐dependent phosphate production, the CD73‐specific inhibitor termed 5’‐(α,β‐methylene) diphosphate ADP analogue (ADPan) was added in some wells before the 5’‐AMP addition. RATregs and RATEVs were incubated without a 5’‐AMP substrate and/or ADPan as controls. After incubation, 80 µL of the supernatant was mixed with the copper‐based colourimetric reagent. After 10 min of gentle shaking, absorbance was measured at 630 nm using a microplate reader (BioTek, VT, USA), and values were compared with a standard curve.

### 2.8 ADO Production

To quantify ADO production, 1 or 2 × 10^5^ RATregs or total RATEVs obtained from 2 × 10^6^ RATregs were incubated in a 96‐well plate with 200 µL of RPMI media supplemented with 10% EVs‐free FBS ADO transporter inhibitor S‐(4‐Nitrobenzyl)‐6‐thioinosine (NBTI) (10 µM, Merck), and adenosine deaminase (ADA) inhibitor erythro‐9‐(2‐Hydroxy‐3‐nonyl)‐adenine hydrochloride (EHNA) (50 µM, Merck). When indicated, 5’‐AMP (5 mM, Merck) was added in the presence or absence of ADPan CD73‐specific inhibitor (1 mM, Merck). After 1 h incubation at 37°C, 10 µL of supernatant was tested for ADO production quantification with the PathHunter ADORA2B assay (DiscoverX, ThermoFisher). The concentration of produced ADO was calculated using a standard curve generated using ADO (Sigma) serially diluted.

### 2.9 T‐Cell Suppression Assay

Erythrocyte‐depleted total leukocytes were obtained from the spleen of C57BL/6 *wild‐type* mice and labelled with 5 µM Cell Trace Violet (CTV, ThermoFisher). Then, T cells were polyclonally activated with soluble anti‐CD3ε (1 µg/mL, clone 145‐2C11, BioXcell). 1 × 10^5^ responder T cells were cultured in the presence of 1 or 2.5 × 10^8^ RATEVs, in RPMI media supplemented with 10% EV‐free FBS. Non‐activated or untreated T cells (exposed to the vehicle PBS) were used as controls. After 72 h, T cells were stained with ZombieDye NIR (Biolegend) viability dye, anti‐CD4 (clone RM4‐5, Biolegend), anti‐CD8 (clone 53–6.7, Biolegend) and CD25 (clone PC61, Biolegend). The effect of RATEVs on T cell proliferation and activation was evaluated by assessing the CTV dilution profile and CD25 expression, respectively. Both parameters were analysed using a FACSCanto II (BD Biosciences) flow cytometer.

### 2.10 Role of CD73 in RATEVs‐Mediated Suppression of CD4^+^ T Cells

The role of CD73 in the immunosuppressive capacity of RATEV was determined by performing a suppression assay on CD4^+^Foxp3^−^CD73^−^ and CD4^+^Foxp3^−^CD73^+^ T cells obtained from C57BL/6 Foxp3^GFP+^ transgenic mice by fluorescence‐assisted cell sorting, using a FACSAria II equipment (BD Biosciences). Then, 1 × 10^5^ responder T cells were activated with plate‐bound anti‐CD3ε (5 µg/mL, clone 145‐2C11, BioXcell) and anti‐CD28 (2 µg/mL, clone 37.51, Biolegend) and cultured in supplemented EV‐free RPMI media in the presence of the ADA inhibitor EHNA (10 µM, Sigma) and ADO transporter inhibitor NBTI (10 µM, Sigma). When indicated, 5'‐AMP (50 µM, Sigma), ADPan or α,β‐methylene adenosine 5′‐diphosphate (APCP) CD73‐specific inhibitors (100 µM, Sigma) and/or RATEVs (1, 2.5, or 5 × 10^8^ particles) were added to the cell cultures. Treatments and reagents were added sequentially in the following order: RATEVs, CD73 inhibitor and 5’‐AMP. After 72 h, the expression of the activation marker CD25 was assessed using a FACSCanto II (BD Biosciences) flow cytometer. In addition, the IL‐17A production was analysed using a specific ELISA kit (ThermoFisher), read in an Elisys Duo ELISA automated analyser (Human Diagnostics, Wiesbaden, Germany).

### 2.11 Induction of Experimental Periodontitis and Treatment With RATEVs

Periodontitis was induced in C57BL/6 mice by tying a 5‐0 silk ligature around the maxillary second molars without causing damage to the periodontal mucosa (de Molon et al. 2018; Rojas et al. 2021). Unligated mice were considered healthy controls (Alvarez et al. 2021; Alvarez et al. 2020; Cafferata et al. 2020). Treatment of periodontitis consisted of inoculation of 1 × 10^8^ (RATEVs‐lo) or 2.5 × 10^8^ (RATEVs‐hi) RATEVs resuspended in 5 µL of filtered PBS at Days 3 and 6 after ligature, injected into the palatal periodontal mucosa adjacent to the second molars using a 26‐gauge Hamilton syringe (Hamilton Company, NV, USA). As treatment control, sham injections with PBS as RATEVs‐free control were used. All these procedures were performed under standard ketamine/xylazine anaesthesia. Selected doses and frequency of RATEV inoculations were determined based on our pilot experiments (data not shown), demonstrating effectiveness in periodontitis control. After 10 days, animals were euthanised by a single overdose of ketamine/xylazine anaesthesia, and samples of maxillae, palatal periodontal mucosa and cervical lymph nodes were harvested for further analysis.

### 2.12 Periodontal Biodistribution of RATEVs

RATEVs were labelled with 71 µM 1,1'‐dioctadecyl‐3,3,3’,3'‐tetramethylindotricarbocyanine iodide (DiR) (Biotum, CA, USA) for 1 h at 37°C. Next, 1 × 10^8^ DiR‐labelled RATEVs were inoculated into the palatal periodontal mucosa adjacent to the second molars, where the periodontal lesions occur. Mice inoculated with RATEVs‐free PBS containing 71 µM DiR were used as negative controls. Immediately after injections, mice were placed in a prone position inside the temperature‐regulated dark chamber of an Odyssey CLx Imaging System (LI‐COR Biosciences, NE, USA). Using an excitation/emission range of 700/780 nm, images showing RATEV‐related fluorescence spatial distribution were taken at the following times: baseline, 30 min, 24 h, 48 h, 72 h and 7 days. After image acquisition, animals were euthanised by a single overdose of ketamine/xylazine anaesthesia and samples of maxillae, liver, kidneys, and cervical and axillary lymph nodes were collected. Images were analysed using the Image Studio v.5.2 software (LI‐COR Biosciences).

### 2.13 Periodontal Immune Response Assessment

Composition of immune cells infiltrating periodontitis‐affected tissues was analysed as previously described (Alvarez et al. 2021; Alvarez et al. 2020). Periodontal mucosa was dissected from the maxilla and immediately digested in RPMI media supplemented with collagenase IV (3.2 mg/mL, Gibco) and DNase (0.15 µg/mL, Merck) for 1 h at 37°C under constant agitation with 50 µL of 0.5 M EDTA being added in the last 5 min of incubation. Suspended cells were then passed through a 70 µm‐size cell strainer. Cervical lymph nodes were directly disaggregated through a 70 µm‐size cell strainer. Periodontal mucosa and cervical lymph node cell quantification was performed using an automated cell counter (Luna II, Logos Biosystems, VA, USA). Separately, 1 × 10^6^ cells were incubated with specific monoclonal antibodies, anti‐CD4, CD8, CD25, CD73, RANKL and CD45 (Table 1) for 30 min at 4°C. Cells were then stimulated with phorbol‐12‐myristate‐13‐acetate (PMA) (50 ng/mL, Merck) and ionomycin (1 µg/mL, Merck), in the presence of 5 µg/mL brefeldin‐A (50 ng/mL, Biolegend), at 37°C for 4 h. Cell fixation and permeabilisation was performed using the Foxp3 Fixation/Permeabilisation Staining Kit (eBioscience, CA, USA), following the manufacturer's instructions, and cells were intracellularly stained overnight using specific monoclonal antibodies to IL‐17A, RANKL and Foxp3 (Table 1). Cell viability was determined using a live/dead kit (LIVE/DEAD Fixable Blue Dead Cell Stain Kit, ThermoFisher). Analysis was performed in a LSRFortessa X‐20 equipment (Beckton Dickinson). Data were analysed using FlowJo v10 software (Beckton Dickinson), applying a sequential gating strategy based on FSC‐A/SSC‐A parameters, FSC‐A/FSC‐H singlet discrimination, live/dead cell staining and CD45, CD4 and CD8 expression. All the experiments were performed separately. As a complement to visualise lymph node data, a Cytobank computational tool, viSNE (visualisation of t‐Stochastic Neighbour Embedding) was used as previously described (Amir el et al. 2013; Campos‐Mora et al. 2019). The expression levels of the markers of interest were expressed with a heat map colour scale.

**TABLE 1:** Monoclonal antibodies for in vivo flow cytometric analysis.

Specificity	Clone	Fluorochrome	Dilution	Supplier	Commercial code
CD4	GK1.5	BV605	1:200	Biolegend	100451
CD8	53‐6.7	BUV396	1:200	BD	563786
CD25	PC61	BV421	1:200	Biolegend	102034
CD45	30‐F11	BV711	1:800	Biolegend	103147
CD73	TY/11.8	PerCP eFluor 710	1:300	Invitrogen	46‐0731‐82
IL‐17A	TC11‐18H10.1	PE Cy7	1:50	Biolegend	506922
RANKL	IK22/5	PE	1:200	Biolegend	510006
Foxp3	150D	Alexa Fluor 488	1:100	Biolegend	320012
Viability	—	BUV496	1:1000	Thermo Fisher	L23105

### 2.14 Alveolar Bone Loss

Tooth‐supporting alveolar bone loss due to periodontitis was analysed as previously described (Alvarez et al. 2021; Alvarez et al. 2020). Briefly, maxillae were mechanically dissected to remove soft tissues, fixed with 10% formalin for 24 h and boiled in distilled water for 5 min to remove any soft tissue remaining. Then, the maxillae were hemisected, rehydrated in a physiological saline solution for 24 h, and their palatal and buccal surfaces were imaged using a Nikon D5600 camera with an 85 mm ED‐VR macro lens (AF‐S DX, Nikon, Japan). Captured images were standardised using the following parameters: f29, S 1/60, ISO 200, flash 1/32 +0.7 and 1:1 magnification and analysed using the ImageJ software (NIH). Alveolar bone loss was quantified as the total area and linear distance of bone resorption as previously described (Alvarez et al. 2021; Alvarez et al. 2020). The resorption area was limited to the following anatomical parameters: CEJ, alveolar bone crest (ABC), mesial surface of the first molar and distal surface of the third molar. The alveolar bone resorption area was quantified by subtracting the mean physiological area determined in the healthy mice. The linear distance of bone resorption was measured from the CEJ to the ABC at nine anatomical periodontal sites. In the first molar: the mesiopalatal cusp, palatal groove, distopalatal cusp, distopalatal groove and distal cusp; in the second molar: the mesiopalatal cusp, palatal groove and distopalatal cusp; and in the third molar: the palatal cusp.

### 2.15 Periodontal Osteoclast Detection

Periodontal osteoclasts expressing the specific marker tartrate‐resistant acid phosphatase (TRAP) were quantified using a previously described protocol (Cafferata et al. 2020). In brief, maxillae were fixed in 4% paraformaldehyde for 24 h, demineralised in 5% EDTA (Merck) for 4 weeks, and processed using a standard histological protocol to be embedded in paraffin. Serial sections of 8 µm were stained using a TRAP histochemical stain kit (Merck) and imaged using an optical microscope (AxioStarPlus, Carl Zeiss, Germany). TRAP^+^ cells with at least three nuclei and close contact with alveolar bone were considered osteoclasts.

### 2.16 Statistical Analysis

Data were statistically analysed using the SPSS v.22.0 software (IBM Corp, NY, USA). The normality of data distribution was established using the Kolmogorov–Smirnov test, and statistical differences were determined using the ANOVA and Bonferroni post hoc tests. The level of significance was set at *α* < 0.05.

## 3 Results

### 3.1 RATregs Release CD73^+^ RATEVs

**FIGURE 1:**
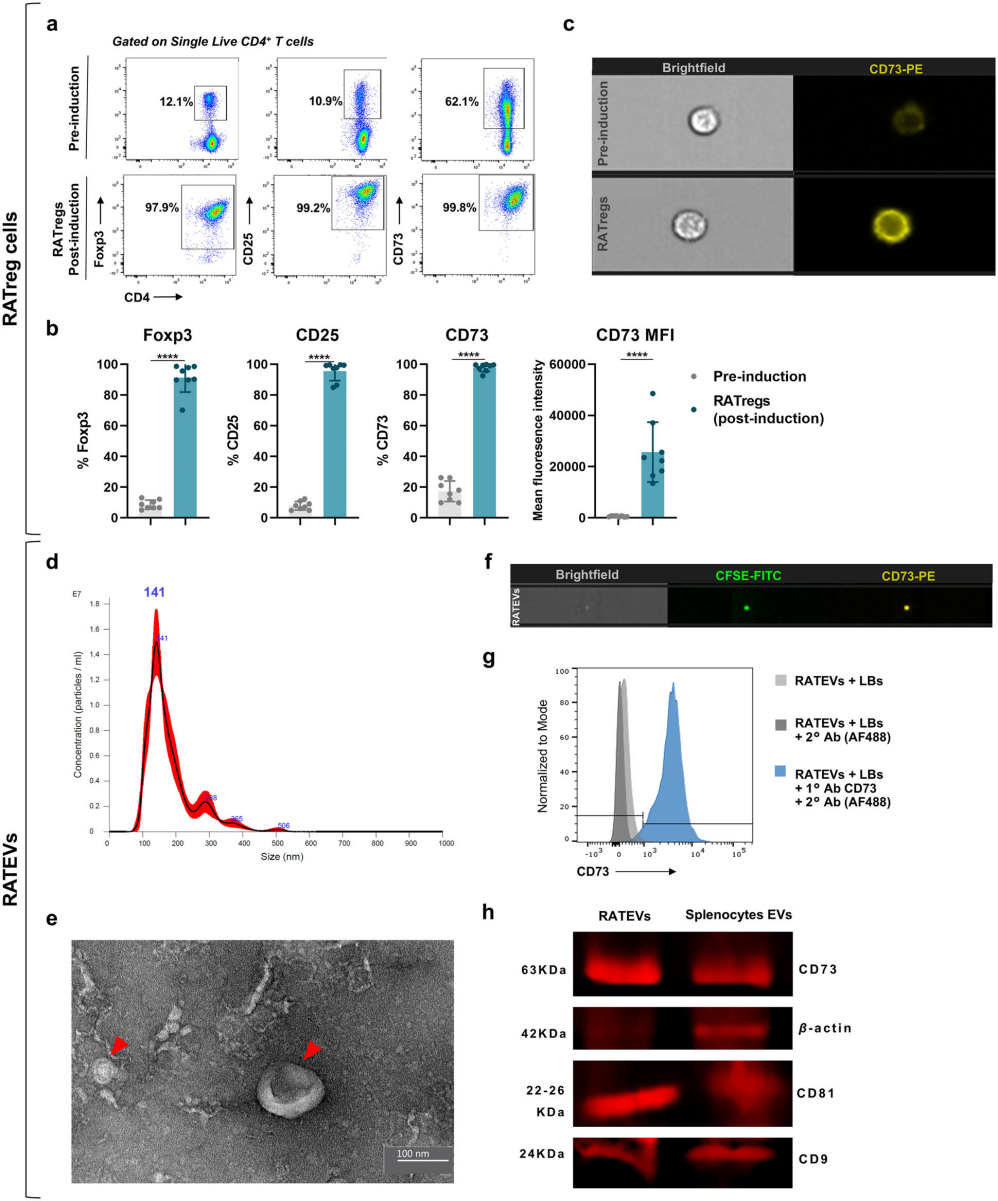
Tregs induced in the presence of retinoic acid (RATregs) highly upregulate CD73 and secrete CD73^+^ EVs (RATEVs). CD4^+^ T cells were magnetically isolated, activated in plate‐bound with anti‐CD3ε and anti‐CD28 (10 and 1 µg/mL, respectively), and exposed to IL‐2 (100 IU/mL), TGF‐β1 (10 ng/mL) and retinoic acid (100 nM) to be differentiated into RATregs. From them, RATEVs were obtained by ultracentrifugation. (a) Representative dot plots showing the Foxp3, CD25 and CD73 expression in non‐induced CD4^+^ T‐cells (upper panel) and RATregs (lower panel) assessed by flow cytometry. (b) The graphs show the quantitative analysis of CD73, Foxp3 and CD25 expression in non‐induced CD4^+^ T cells and RATregs induced by polarising cytokines and retinoic acid. (c) RATreg CD73 expression and cellular localisation analysis by imaging flow cytometry. (d) Characterisation of RATEVs size and relative concentration by nanoparticle tracking analysis (NTA). (e) Size and morphology of RATEVs by transmission electron microscopy (TEM). (f) RATEVs CD73 expression assessment by imaging flow cytometry. (g) RATEV CD73 expression assessment by latex beads‐assisted flow cytometry. The blue histogram represents bead‐bound RATEVs incubated with anti‐CD73 and anti‐mouse Alexa Fluor 488 antibodies. Beads‐bounded RATEVs (light grey histogram) and beads‐bounded RATEVs incubated only with the secondary antibody (dark grey histogram) were used as controls. (h) Immunoblot analysis for CD73, β‐actin, CD81 and CD9 in RATEVs and EVs obtained from erythrocyte‐depleted splenocytes. ****p < 0.001.

From C57BL/6 Foxp3^GFP+^ mice, a highly pure population (>95%) of CD4^+^ T lymphocytes was obtained (Figure S1a). These cells were differentiated into RATregs using a combination of polarising cytokines and RA, and the resulting phenotype was analysed by flow cytometry (Figure 1a), with the gating strategy detailed in Figure S1b. Following induction, Foxp3, CD25 and CD73 expression were significantly upregulated (Figure 1b), indicating that RATregs acquired the characteristic CD4^+^Foxp3^+^CD25^high^ regulatory phenotype, along with elevated CD73 levels. Given the marked upregulation of CD73, we carried out a comparative quantitative analysis of CD73 expression across different Treg subsets: nTregs, iTregs and RATregs. This analysis revealed that RATregs exhibited the highest levels of CD73 expression, as determined by mean fluorescence intensity (MFI) (Figure S1c). Furthermore, CD73 was predominantly localised on the cell surface of RATregs, closely associated with the plasma membrane (Figure 1c), mirroring the distribution pattern observed for CD4 and CD25 (Figure S1d).

From RATregs conditioned media, RATEVs were isolated and their concentration and size distribution were analysed by NTA (Figure 1d). Most particles ranged from 100 to 200 nm, and the mean size was 141 nm (±71 nm). In addition, three small fractions were observed, ranging from around 288–506 nm. These distinct RATEV sizes were ratified by TEM (Figure 1e). To demonstrate that RATEVs maintain the CD73 cargo observed in RATregs, the presence of CD73 on RATEVs was confirmed using distinct experimental approaches. CD73 enrichment was demonstrated using imaging flow cytometry analysis (Figure 1f). Bead‐assisted flow cytometry analysis showed that the CD73‐related fluorescence signal was only detected on RATEV samples, being undetected in controls (Figure 1g). Lastly, Western blot analysis confirmed CD73 presence on RATEVs (Figure 1h). As a positive control, the presence of CD73, CD81 and CD9 was also analysed in EVs obtained from total splenocytes. Whole Western blot membranes are shown in Figure S2a. Taken together, these results indicate that purified RATEVs are CD9^+^CD81^+^ EVs loaded with the regulatory ectoenzyme CD73.

### 3.2 CD73 From RATregs and RATEVs Possesses AMPase Activity and Catalyses ADO and Inorganic Phosphate Production

**FIGURE 2:**
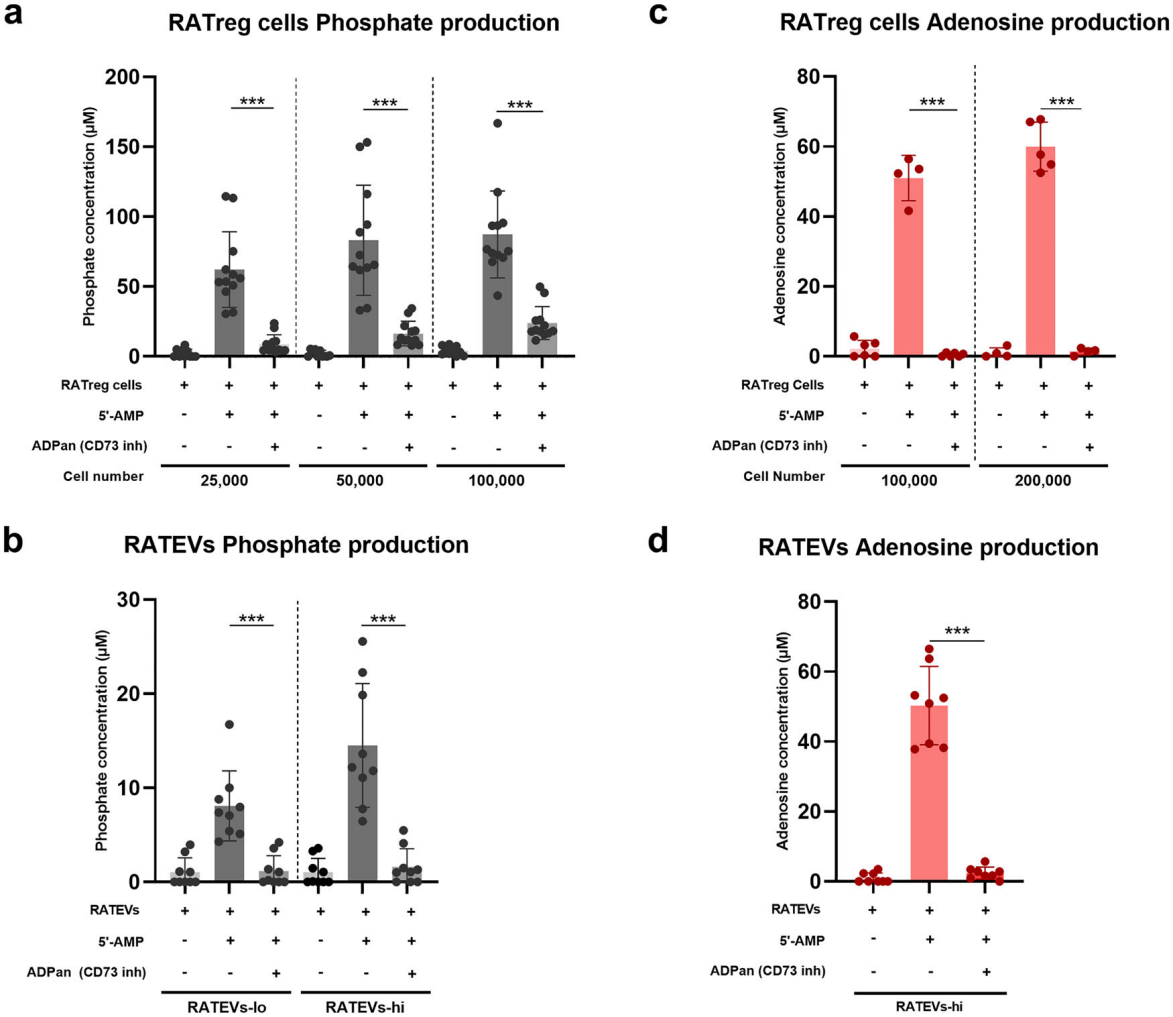
In RATregs and RATEVs, CD73 exerts AMPase activity and catalyses ADO and phosphate production. The CD73‐mediated AMPase activity in RATregs and RATEVs was evaluated by assessing inorganic phosphate and adenosine (ADO) production. (a) 2.5, 5, or 10 × 10^4^ RATregs or (b) RATEVs obtained from 1 or 2 × 10^6^ RATregs were resuspended in phosphate‐free buffer and incubated alone or in the presence of 5’‐AMP. In some conditions, the CD73‐specific inhibitor termed 5’‐(α,β‐methylene) diphosphate ADP analogue (ADPan) was added. To quantify phosphate levels, values were compared to a standard curve. Data represent the amount of inorganic phosphate produced over background (RATregs or RATEVs incubated in the absence of 5’‐AMP) and are shown as mean±SD of five samples pooled from five independent experiments. Supernatant ADO levels produced by (c) 1 or 2 × 10^5^ RATregs and (d) RATEVs obtained from 2 × 10^6^ RATregs were measured in samples incubated alone or in the presence of 5’‐AMP. In some conditions, ADPan (CD73 inhibitor) was added. Control conditions included culture medium alone, medium with AMP, or AMP and CD73 inhibitor (data not shown). To quantify the ADO levels, values were compared to a standard curve. ***p < 0.001.

CD73 exerts 5’‐AMP‐hydrolytic (AMPase) activity and produces inorganic phosphate and ADO, a well‐known CD73‐dependent immunoregulatory mechanism (Antonioli et al. 2013; Chen et al. 2023). Considering that both RATregs and RATEVs express CD73, their AMPase activity was analysed following exposure to exogenous 5’‐AMP as substrate in the presence or absence of a CD73 inhibitor (specified in the methods section). As expected, after AMP exposure, an increase in phosphate production was observed in RATregs (62.23±27.07 µM; 83.22±39.44 µM and 87.37±31.07 µM for 2.5, 5 and 10 × 10^4^ cells, respectively) and RATEVs (8.08±3.73 µM and 14.5±6.58 µM for RATEVs obtained from 1 or 2 × 10^6^ RATregs, respectively), an effect not observed in the absence of RATregs or RATEVs. When CD73 was neutralised using its specific inhibitor, phosphate production significantly decreased, which was ratified using different RATregs (8.8±6.74 µM, 16.4±8.85 µM and 23.9±11.86 µM, respectively) (Figure 2a) and RATEVs quantities (1.15±1.64 µM and 1.61±1.93 µM) (Figure 2b). More importantly, ADO production was observed after AMP exposure by both CD73‐bearing RATregs (42.42±19.96 µM and 59.97±7.02 µM for 1 and 2 × 10^5^ cells) (Figure 2c) and RATEVs (50.3±11.2 µM for RATEVs obtained from 2 × 10^6^ RATregs) (Figure 2d). This effect was significantly repressed in the presence of the CD73 inhibitor (0.46±0.52 µM and 1.37±1 µM for 1 and 2 × 10^5^ cells and 2.3±1.7 µM for RATEVs obtained from 2 × 10^6^ RATregs), reaching similar levels to those observed in the negative controls. These results suggest that CD73 present in 2.5–10 × 10^5^ RATregs or RATEVs isolated from 2 × 10^6^ RATregs produce comparable amounts of inorganic phosphate and ADO after 5’‐AMP exposure. However, although we can estimate the number of RATEVs secreted by the RATregs, the number of CD73 molecules per vesicle is unknown, which makes direct comparisons of AMPase activity between RATEVs and RATregs imprecise. It is important to note that although different concentrations of the 5’‐AMP substrate were used in the phosphate and adenosine assays, the levels of product generated were similar. This suggests that the enzyme was likely operating near its maximum catalytic capacity under both conditions, consistent with saturation kinetics. These observations confirm that CD73 present on RATregs and RATEVs has AMPase activity and catalyses the production of ADO, a nucleoside with strong immunosuppressive capacity.

### 3.3 RATEVs Suppress T‐Cell Proliferation and Activation

**FIGURE 3:**
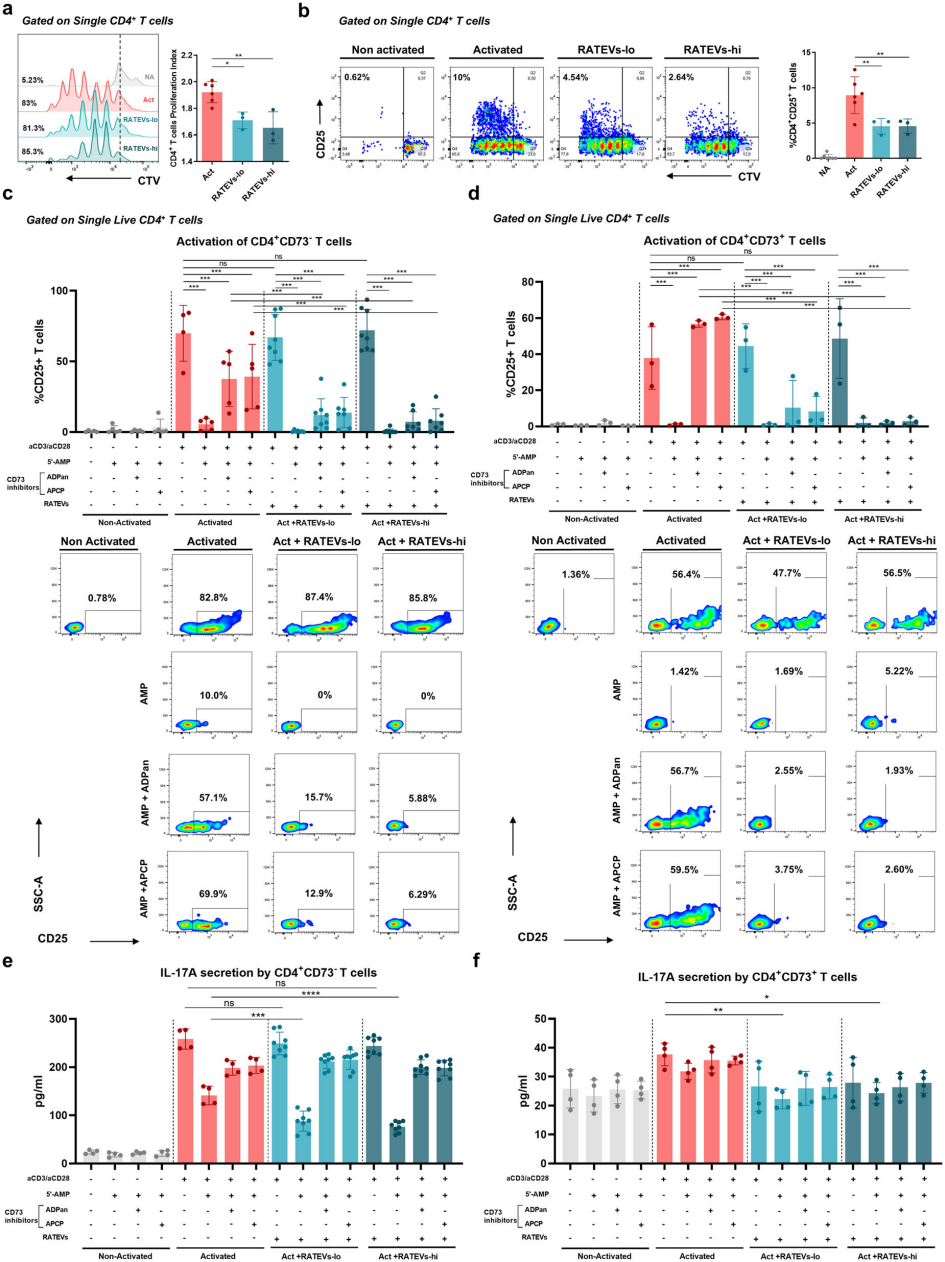
CD73‐mediated AMPase activity support RATEV immunosuppressive capacity over CD4^+^ T cells. Erythrocyte‐ depleted splenocytes derived T cells labelled with Cell Trace Violet (CTV) were activated in vitro with soluble anti‐CD3ε (1 µg/mL) and treated with RATEVs (1 and 2.5 × 10^8^ particles, termed RATEVs‐lo and RATEVs‐hi, respectively) for 72 h. Non‐activated (NA) or activated (Act) untreated cells (exposed to the vehicle) were defined as negative and positive activation controls, respectively. (a) Representative histograms that show CD4^+^ T cell proliferation (CTV dilution) in the following conditions: Non‐activated (grey line), untreated activated (red line) and RATEV‐treated activated (light and dark blue lines) cells. Each histogram peak represents a proliferation cycle in which parent cells are divided. The graph shows the proliferation index values as mean±SD. (b) Representative dot plots and graphs of the mean percentage for activated (CD25^+^) CD4^+^ T cells. (c) CD4^+^CD73^−^ or (d) CD4^+^CD73^+^ responder T cells obtained from C57BL/6 Foxp3^GFP+^ transgenic mice and isolated by cell sorting were activated with anti‐CD3ε (5 µg/mL) and anti‐CD28 (2 µg/mL) antibodies, in the presence of EHNA (10 µM) and NBTI (10 µM) inhibitors, and incubated with or without 5’‐AMP (50 µM), RATEVs (1 or 2.5 × 10^8^ particles, termed RATEVs‐lo and RATEVs‐hi, respectively), and/or ADPan or APCP CD73 inhibitors (100 µM). T‐cell activation was evaluated by quantifying the CD25 expression using flow cytometry. Immediately below the charts, representative smoothed dot plots are shown for each condition. The assay was performed in duplicate for each condition, and graphs show data presented as CD25^+^ T cells mean±SD percentage. In (e) CD4^+^CD73^−^ and (f) CD4^+^CD73^+^ responder T cells, activated and treated following the protocol described for (c) and (d), the IL‐17A production was analysed. Data are presented as mean±SD pg/mL. *p < 0.05, **p < 0.01, ***p < 0.001. ns = non‐significant.

CD73‐mediated ADO production has been consistently related to the immunoregulatory capacity of murine Tregs, particularly to the suppression of T‐cell responses (de Oliveira Bravo et al. 2016). Consequently, we investigated whether CD73‐bearing RATEVs exert immunosuppression by targeting the proliferation and/or activation of CD4^+^ T cells. Considering the reported similarities between the frequencies of spleen and gingival mucosa‐derived immune cells (Monasterio et al. 2020), we mounted a suppression assay using erythrocyte‐depleted splenocytes. In this assay, T cells were polyclonally activated in the presence of increasing concentrations of RATEVs. In this context, RATEVs significantly suppressed CD4^+^ T cell proliferation compared to untreated T cells (1.92±0.08 vs. 1.72±0.06 and 1.64±0.12 proliferation index for untreated vs. 1 and 2.5 × 10^8^ RATEVs, respectively) (Figures 3a **and** S3a). These results align with a previous report using parental RATregs (Benson et al. 2007). In addition, RATEVs significantly reduced T cell activation, as demonstrated by downregulation of CD4^+^CD25^+^ T cells once exposed to different RATEV quantities (8.95±2.39% vs. 4.40±0.80% and 4.35±0.77% for untreated vs. 1 and 2.5 × 10^8^ RATEVs) (Figures 3b **and** S3b).

### 3.4 CD73‐Mediated AMP Hydrolysis Drives CD4^+^ T‐Cells Suppression

Having confirmed the presence and AMPase activity of CD73 on RATEVs, we next determined whether the suppressive capacity of RATEVs was CD73‐dependent. To address this, we conducted a suppression assay using highly pure (>99%) CD4^+^CD73^−^ or CD4^+^CD73^+^ sorted T cells as responder cells (Figure S3c,d). We initially assessed the effect of endogenous CD73 on T‐cell activation, measured as increasing CD25 expression, in the presence of exogenous AMP and using two types of CD73 inhibitors in the absence of RATEVs (Figure 3c, red bars). Polyclonal activation induced a significant CD25 upregulation in CD4^+^CD73^−^ (69.8±19.7%) (Figure 3c) and CD4^+^CD73^+^ (39.3±24.2%) (Figure 3d) T cells, compared to non‐activated cells (0.4±0.6% and 0.9±0.6% for CD4^+^CD73^−^ and CD4^+^CD73^+^ T cells, respectively). This effect was not observed in the presence of AMP, in which CD25 expression was significantly reduced in both T‐cell subtypes (5.4±4% and 0.9±0.7% for CD4^+^CD73^−^ and CD4^+^CD73^+^, respectively). Neutralisation of CD73 activity largely restored CD25 expression on CD4^+^CD73^−^ T cells after activation (37.5±19.4% and 39.22±22.8% for ADPan and APCP CD73 inhibitors, respectively) (Figure 3c). Interestingly, on CD4^+^CD73^+^ T cells, CD73 inhibition induced even higher CD25 levels than those detected at baseline (57.7±1.3% and 60.9±1.9% for AMP plus ADPan and APCP CD73 inhibitors vs. 39.3±24.2% on untreated activated cells) (Figure 3d). Having noted that CD73^+^ T‐cell activation was abrogated by AMP exposure, we analysed whether TCR stimulation could have induced CD73 expression on CD4^+^CD73^−^ T cells, leading to ADO generation from the exogenous AMP, leading to activation inhibition. Figure S3e shows that αCD3ε/αCD28‐activated CD4^+^CD73^−^ T cells upregulated CD73 expression (from 0.42±0.6% in the absence of polyclonal stimulation to 43.22±13.4% after activation), making them susceptible to ‘self‐suppression’ in the presence of AMP. Conversely, as expected, >90% of CD4^+^CD73^+^ T cells expressed CD73 in all experimental conditions (Figure S3f), explaining activation suppression upon AMP addition. Altogether, these data demonstrated that CD4^+^CD73^−^ and CD4^+^CD73^+^ T‐cell activation decreased in the presence of AMP substrate, which was reversed when CD73 activity was blocked.

### 3.5 CD73 Plays a Relevant Role in RATEV‐Mediated Suppression of CD4^+^ T‐Cells

Having characterised the effect of exogenous AMP exposure on T‐cell activation, we further analysed the effect of RATEVs on CD4^+^CD73^−^ and CD4^+^CD73^+^ T‐cell activation in the same experimental settings previously described. As expected, RATEVs suppressed CD25 expression on both CD4^+^CD73^−^ (Figure 3c) and CD4^+^CD73^+^ T cells (Figure 3d) following activation in the presence of AMP (0.4±0.6% and 0.7±1.5% for RATEVs‐lo and RATEVs‐hi on CD73^−^ T cells and 1.0±0.6% and 1.9±2.8% for RATEVs‐lo and RATEVs‐hi on CD73^+^ T cells, respectively). When CD73 activity was inhibited, CD4^+^CD73^−^ T cells exposed to RATEVs showed a partial recovery of CD25 expression (12.05±11.5% and 7.22±7% for RATEVs‐lo and RATEVs‐hi in the presence of ADPan CD73 inhibitor and 11.9±11 and 4.33±4.4% for RATEVs‐lo and RATEVs‐hi in the presence of APCP CD73 inhibitor) (Figure 3c). This recovery was less pronounced than the one observed in the absence of RATEVs when CD73 activity was inhibited (37.5±19.4% and 39.22±22.8% for ADPan and APCP, respectively) (Figure 3c, third and fourth red column). On CD4^+^CD73^+^ T cells exposed to AMP plus RATEVs, reduced CD25 expression was also observed when CD73 activity was inhibited (10.4±15.1% and 2±1% for RATEVs‐lo and RATEVs‐hi in the presence of APDan and 8.3±8.3% and 2.9±2.3% for RATEVs‐lo and RATEVs‐hi in the presence of APCP, respectively). Notably, with RATEVs‐hi treatment, CD73 inhibitors did not restore CD4^+^CD73^+^ T cell activation (2±1% for ADPan and 2.9±2.3% for APCP) (Figure 3d). Conversely, inhibition of CD73 before AMP exposure in the absence of RATEVs resulted in a marked upregulation of CD25 expression, even higher than basal activation levels (57.7±1.3% and 60.9±1.9% for ADPan and APCP, respectively) (Figure 3d). These results suggest that the AMPase activity on CD73^+^ T cells causes ADO production. In addition, CD73 expressed on RATEVs leads to robust suppression of T‐cell activation, which could not be reversed by the CD73 inhibition strategies employed in this study.

### 3.6 CD73‐Mediated RATEVs Suppression of IL‐17A Selectively Targets CD4⁺CD73⁻ T Cells

To ratify the role of CD73 in the suppressive capacity of the RATEVs, we quantified the IL‐17A levels produced by the responder CD4^+^CD73^−^ and CD4^+^CD73^+^ T cells. IL‐17A production was negligible in resting CD4^+^CD73^−^ T cells and upregulated upon polyclonal stimulation (Figure 3e). Importantly, AMP exposure either alone or in combination with RATEVs resulted in reduced IL‐17A production in activated CD4^+^CD73^−^ T cells, confirming the requirement of AMP for RATEVs to suppress these cells. Unlike CD25 expression, IL‐17A secretion was restored when CD73 was inhibited. In contrast, CD4^+^CD73^+^ T cells displayed a different response (Figure 3f). First, polyclonal activation induced only a slight increase in IL‐17A production compared to resting T cells. IL‐17A production was significantly reduced only in response to RATEVs combined with AMP, and its secretion was not restored upon CD73 inhibition. These results suggest that CD73 AMPase activity is more relevant in suppressing CD4^+^CD73^−^ than CD4^+^CD73^+^ T cells. These findings demonstrate the important role of CD73 in mediating the immunosuppressive effects of RATEVs on CD4^+^ T cells.

### 3.7 CD73‐Bearing RATEVs Suppress T‐Cell Osteolytic Phenotype During Periodontitis

**FIGURE 4:**
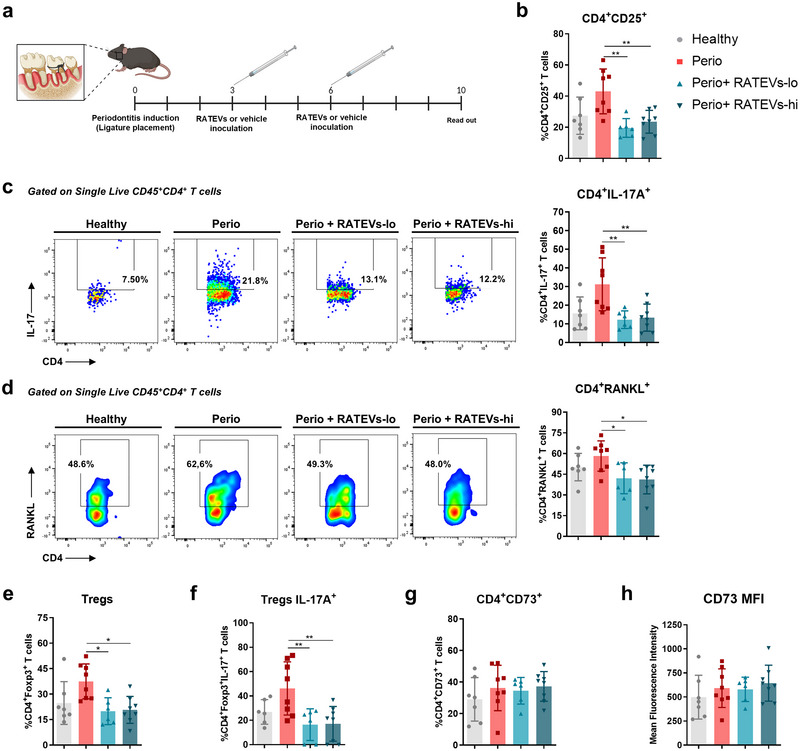
RATEVs suppress CD4+ T‐cell immune responses in periodontitis‐affected periodontal tissues. (a) Schematic representation of the experimental design showing the periodontitis induction model and RATEVs inoculation scheme. Animals were treated with RATEVs at two concentrations (1 and 2.5 × 10^8^ particles, termed RATEVs‐lo and RATEVs‐hi, respectively). At Day 10, periodontal tissue samples were isolated and processed for flow cytometry analysis. (b) Graph showing the frequency of CD4^+^CD25^+^ T cells. (c) Representative dot plots and graphs showing the frequency of CD4^+^IL‐17A^+^ T cells. (d) Representative dot plots and graphs showing the frequency of CD4^+^RANKL^+^ T cells. (e) Graph showing the frequency of total CD4^+^Foxp3^+^ Tregs. (f) Graph showing the frequency of CD4^+^Foxp3^+^IL‐17A^+^ Tregs. (g) Graph showing the frequency of CD4^+^CD73^+^ T cells. (h) CD73 MFI on CD4^+^ T cells. Data are expressed as mean±SD. *p < 0.05, **p < 0.01.

Periodontitis is characterised by a Th17/Treg imbalance, where Th17 cells produce high levels of IL‐17A and RANKL. In this setting Tregs display a decreased suppressive capacity in favour of producing IL‐17A (Alvarez et al. 2019; Tsukasaki et al. 2018). Having determined that RATEVs suppress CD4^+^ T‐cell proliferation and activation in vitro, we analysed whether RATEVs could serve as a therapeutic agent, by suppressing T‐cell osteolytic function, in a murine experimental model of periodontitis (Figure 4a). First, we confirmed the periodontal distribution of CD73‐bearing RATEVs upon local injection in healthy animals (Figure S4a). Using full‐body imaging analysis, RATEVs were detected in the periodontal tissues for up to 24 h post‐inoculation. Nevertheless, when the organs were analysed ex vivo, a signal was still detected in the periodontal tissues up to 7 days post‐inoculation (Figure S4b), likely reflecting tissue retention of the fluorescent dye originally associated with the EVs. This retention may result from the uptake of labelled EVs or diffusion of free dye into resident cells, a phenomena that have been extensively reported in the literature (Liu et al. 2022; Progatzky et al. 2013). Subsequently, the effect of RATEVs treatment on periodontal tissues‐infiltrating CD4^+^ T cells was characterised by flow cytometry following the gating strategy detailed in Figure S5a. Periodontitis‐affected mice (Perio mice) treated with RATEVs showed a reduction in the frequencies of CD4^+^CD25^+^ T cells compared to untreated mice (19.58±6.01% and 27.63±14.07% for RATEVs‐lo and RATEVs‐hi vs. 43.05±14.39% for untreated Perio mice), suggesting a decreased CD4^+^ T‐cell activation in the periodontitis affected tissue (Figure 4b). Remarkably, RATEVs treatment triggered a significant reduction in the frequencies of IL‐17A‐producing CD4^+^ T cells in periodontal lesions compared to untreated controls (12.24±4.72% and 13.42±6.8% for RATEVs‐lo and RATEVs‐hi vs. 31.16±14.18% for untreated Perio mice) (Figure 4c), which was complemented by a significant decrease in the frequencies of RANKL‐expressing CD4^+^ T cells (42.07±11.22% and 44.97±14.96% for RATEVs‐lo and RATEVs‐hi vs. 58.21±11.01% for untreated Perio mice) (Figure 4d). When CD4^+^Foxp3^+^ Tregs were analysed, RATEVs treatment also reduced their frequencies in periodontitis‐affected tissues (19.92±7.9% and 24.39±13.29% for RATEVs‐lo and RATEVs‐hi vs. 37.49±10.29% for untreated Perio mice) (Figure 4e); in particular, a significant decrease of IL‐17A‐producing Tregs was detected (24.67±4.26% and 28.05±6.77% for RATEVs‐lo and RATEVs‐hi vs. 46.21±21.86% for untreated Perio mice) (Figure 4f). Taking all these results together, we can conclude that RATEV treatment suppresses not only IL‐17A and RANKL‐producing CD4^+^ T cells but also IL‐17A‐producing Tregs with osteoclastogenic potential during periodontitis.

### 3.8 CD73‐Bearing RATEVs Treatment Triggers CD73 Upregulation on T Cells

**FIGURE 5:**
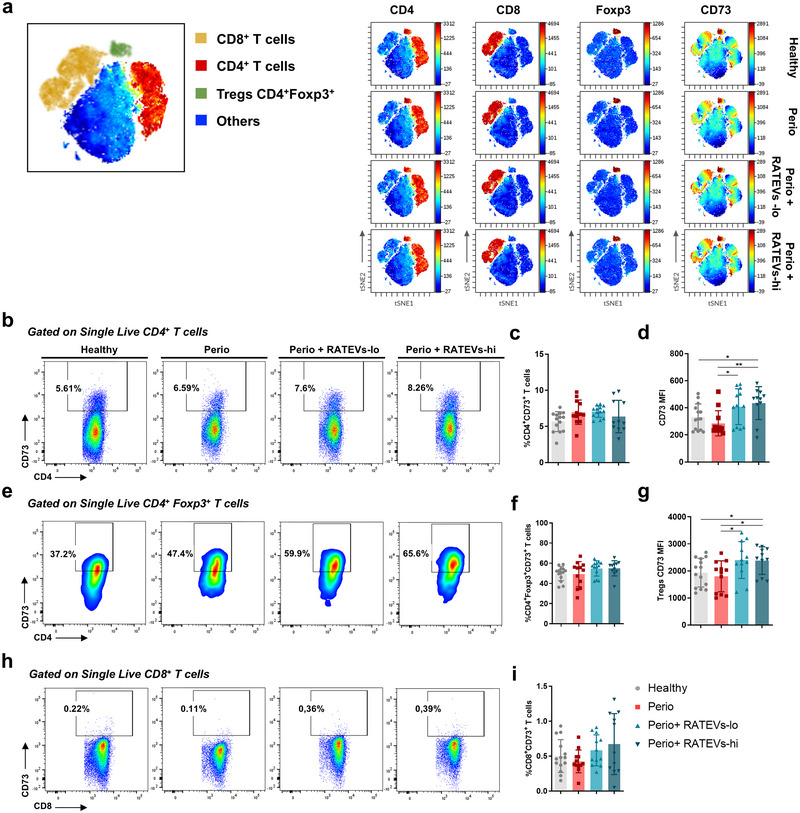
RATEVs upregulate CD73 expression in T cells infiltrating cervical lymph nodes that drain periodontitis‐affected periodontal tissues. (a) Schematic viSNE map (left figure) showing the topographical location of cell populations in which CD73 expression was assessed. Subpopulations are represented using colour‐coding (CD4^+^ T cells, CD8^+^ T cells, Tregs and other immune cells). Representative maps from the viSNE analysis of 1 × 10^4^ cells, where the different lineage expression (CD4 and CD8 T cells) and phenotypic markers Foxp3 and CD73 were analysed and expressed in a heat map‐like colour scale (right panel). The viSNE maps show in red the spatial location of three cell populations: CD4^+^ T cells (first column), CD8^+^ T cells (second column) and CD4^+^Foxp3^+^ Tregs (third column). CD73 expression levels in the aforementioned cells are shown in the fourth column. (b) Representative dot plots showing the frequency of CD4^+^CD73^+^ T cells assessed by flow cytometry. (c) Graph showing the frequency of CD4^+^CD73^+^ T cells. (d) Graph showing CD73 expression in CD4^+^ T cells measured as MFI. (e) Representative dot plots showing the frequency of CD4^+^Foxp3^+^CD73^+^ Tregs. (f) Graph showing the frequency of CD4^+^Foxp3^+^CD73^+^ Tregs. (g) Graph showing CD73 expression in CD4^+^Foxp3^+^ Tregs measured as MFI. (h) Representative dot plots showing the frequency of CD8^+^CD73^+^ T cells. (i) Graph showing the frequency of CD8^+^CD73^+^ T cells. Data are expressed as mean±SD. *p < 0.05, **p < 0.01. ns = non‐significant.

Considering the relevant role that CD73 plays in local immune response modulation, we analysed whether CD73‐bearing RATEVs treatment triggered any changes in CD73 expression on target T‐cells that infiltrated periodontal lesions and/or drain cervical lymph nodes, where they recognise the cognate periodontitis‐related antigens by interacting with antigen‐presenting cells. Within periodontal lesions, no differences in the frequencies of CD4^+^CD73^+^ T cells (Figure 4g) or CD73 MFI signal (Figure 4h) were observed between treated and untreated periodontitis mice. CD73 expression was then analysed on total CD4^+^ T cells, CD4^+^Foxp3^+^ Tregs and CD8^+^ T cell populations (Figure S5b) within the draining cervical lymph nodes, using the high dimensionality t‐SNE method, whereby CD73‐expressing T cells were visualised using viSNE maps and a heat map‐like colour scale (Figure 5a) and conventional methods (unstained control is shown in Figure S5c). When CD4^+^ T cells were analysed, no differences in the frequencies of CD4^+^CD73^+^ T cells were observed (Figure 5b,c). However, a significantly higher CD73 MFI signal was observed in CD4^+^ T cells infiltrating periodontitis‐affected mice treated with RATEVs compared to untreated controls (Figure 5b,d). This effect was also detected on CD4^+^Foxp3^+^ Tregs, in which the total frequency of Tregs expressing CD73 showed no differences among the experimental conditions (Figure 5e,f), but a significantly higher CD73 MFI signal was observed in RATEVs‐treated mice as compared to untreated controls (Figure 5e,g). When CD8^+^ T cells were analysed, no differences in the frequencies of CD8^+^CD73^+^ T cells were observed (Figure 5h,i). In summary, periodontitis‐affected mice treated with RATEVs display T‐cell subsets with increased CD73 expression, suggesting that RATEV treatment could act in a CD73‐dependent manner.

### 3.9 CD73‐Bearing RATEVs Treatment Reduces Osteoclastogenesis and Ameliorates Bone Loss During Periodontitis

**FIGURE 6:**
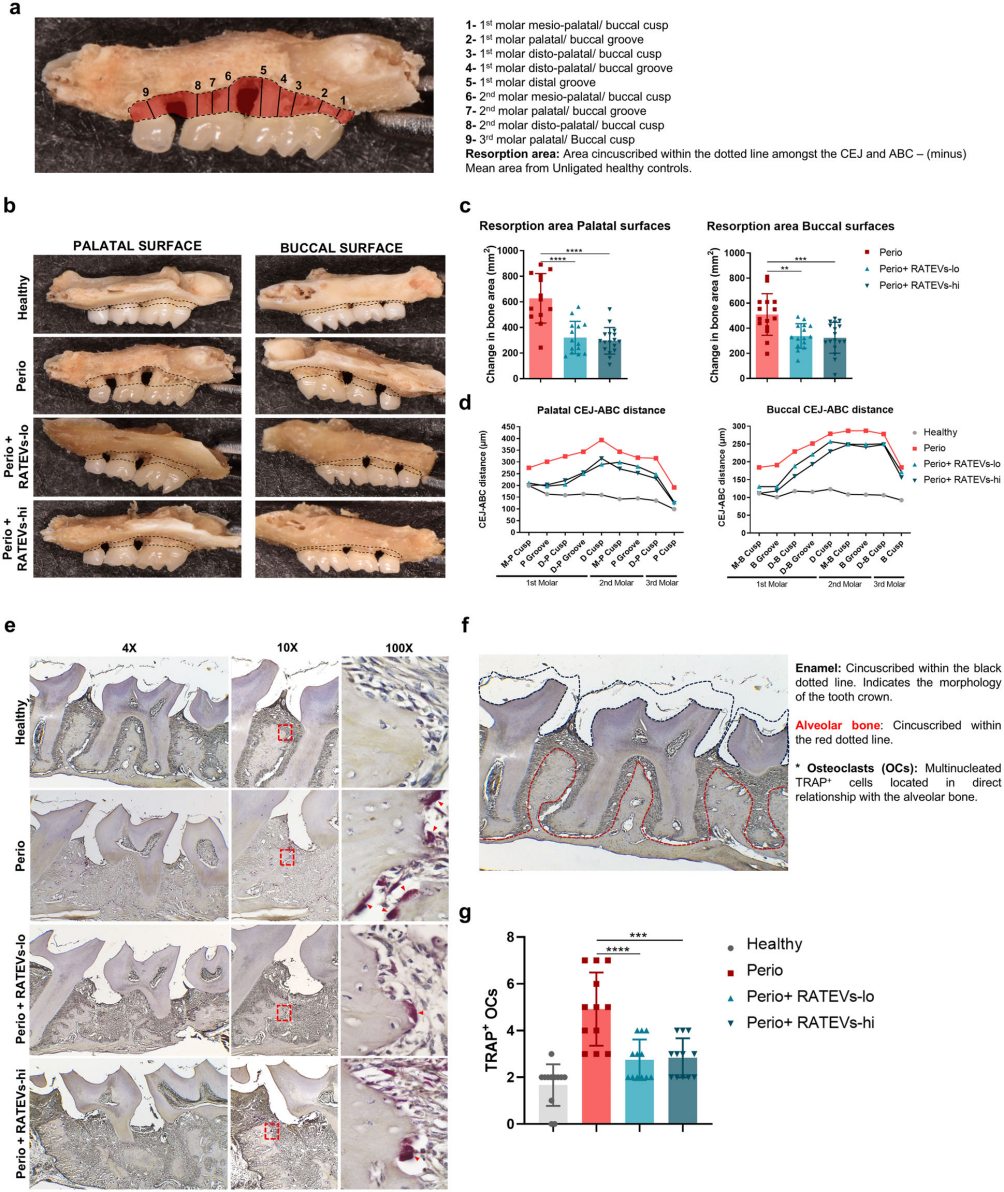
RATEVs ameliorate bone resorption during periodontitis. (a) Illustrative figure showing how measurements for the distance between the cement‐enamel junction (CEJ) and alveolar bone crest (ABC) were performed to analyse tooth‐supporting bone loss. The lines correspond to the CEJ‐ABC distance measured in the three maxillary molars. (b) Representative maxillary alveolar bone photographs for each experimental condition. The dotted black line corresponds to the area delimited by the CEJ, ABC, mesial surface of the first molar and distal surface of the third molar. (c) Bone resorption area quantification was evaluated on the palatal (left) and buccal (right) surfaces. Data are expressed as the change in the bone area after subtracting the mean area value obtained from the unligated healthy controls used as a reference. (d) CEJ‐ABC linear distance quantification evaluated in the nine anatomic sites of the maxilla palatal, and buccal surfaces. (e) Representative images of TRAP^+^ osteoclast (OCs) detection in histologic samples for each condition. Acquired at 4× (left column), 10× (middle column) and 100× (right column) magnification. The red dashed line box delimits the area selected for the 100× magnification, and the red arrowheads show TRAP^+^ osteoclasts in direct contact with alveolar bone. (f) Schematic representation that illustrates the teeth and alveolar bone anatomy for OCs detection. (g) Number of TRAP^+^ osteoclasts detected for each experimental condition. **p < 0.01, ***p < 0.001, ****p < 0.0001.

CD4^+^ T cells are key determinants of tooth‐supporting bone resorption during periodontitis. Given our previous findings, we assessed whether RATEV treatment ameliorated periodontal bone loss by measuring the CEJ‐ABC distance (Figure 6a) and area (Figure 6b) of the tooth‐supporting bone. In untreated periodontitis‐affected mice, an extensive area of bone loss was observed, with marked furcation lesions in the most severe cases (Figure 6b). Along the palatal (321±160.9 mm^2^ and 295.8±103.54 mm^2^ for RATEVs‐lo and RATEVs‐hi vs. 627.5±321.5 mm^2^ for untreated Perio mice) and buccal (338±97.9 mm^2^ and 323.44±123.4 mm^2^ for RATEVs‐lo and RATEVs‐hi vs. 509.4±165.5 mm^2^ for untreated Perio mice) surfaces, a significant reduction in the bone resorption area was observed in response to the RATEVs treatment (Figure 6c). Likewise, a decreased CEJ‐ABC distance was detected upon RATEV treatment within all the analysed periodontal sites compared to untreated periodontitis (Figure 6d). Periodontal osteoclast detection was examined to support the morphometric findings demonstrating bone loss amelioration when periodontitis‐affected mice were treated with RATEVs (Figure 6e,f). Compared to healthy mice, increased TRAP^+^ osteoclasts, directly related to bone resorption lacunae, were observed in untreated periodontitis‐affected mice (Figure 6g). Nonetheless, osteoclast detection significantly decreased in the RATEVs‐treated periodontitis mice (Figure 6g). These results demonstrated that RATEVs inhibit osteoclastogenesis and ameliorate alveolar bone loss during experimental periodontitis.

## 4 Discussion

**FIGURE 7:**
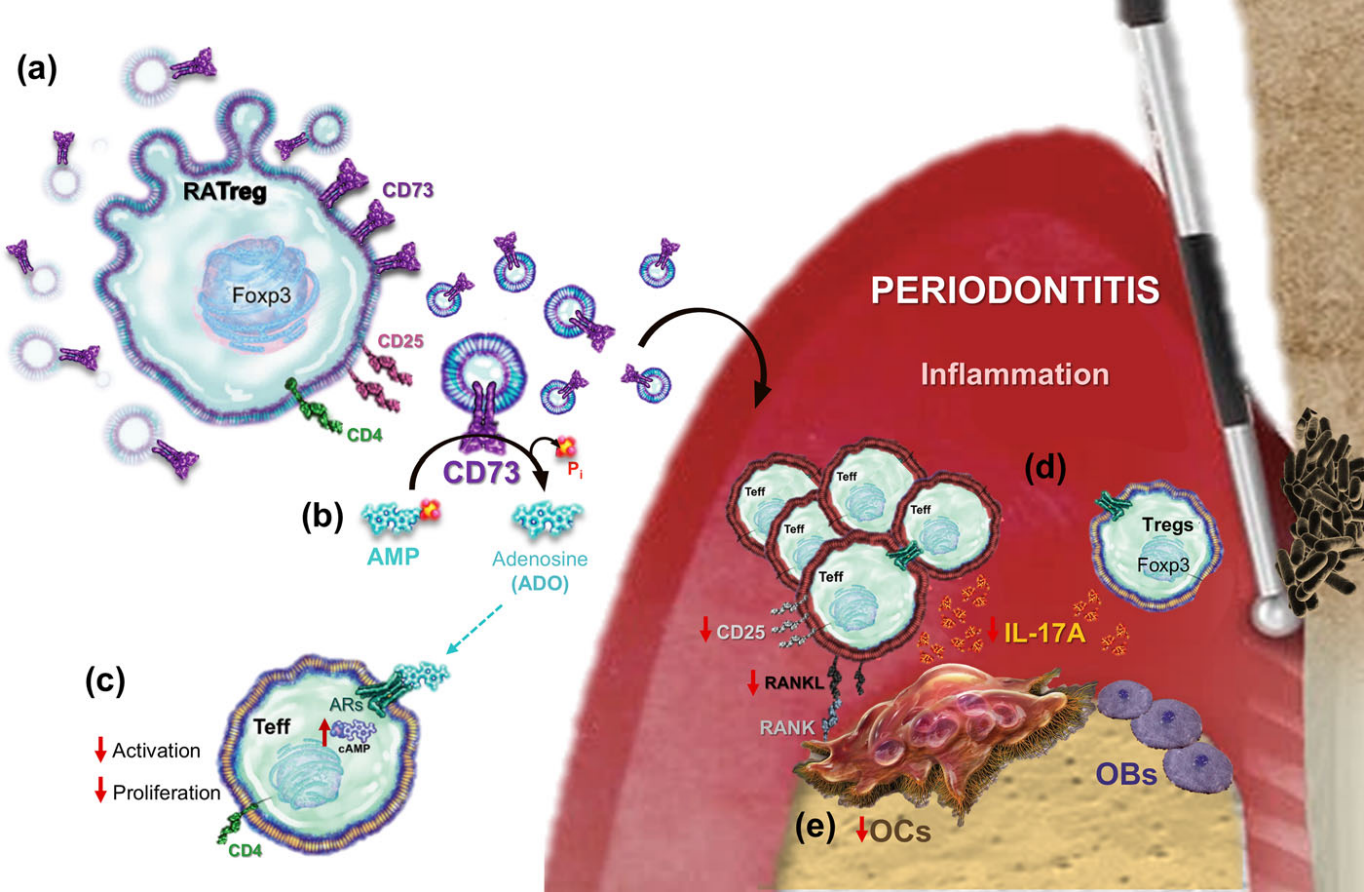
Graphical summary. (a) RATregs express high levels of CD73 and (b) secrete CD73^+^ RATEVs capable of hydrolysing exogenous AMP into the immunosuppressive ADO. (c) RATEVs suppressed CD4^+^ effector T cells in a CD73/AMP/ADO‐dependent manner. (d) The local administration of RATEVs into periodontitis lesions controlled the inflammatory and osteolytic response by suppressing activated (CD25^+^), IL‐17A‐producing and RANKL‐expressing CD4^+^ T cells. (e) RATEV treatment decreased the number of bone‐resorbing osteoclasts and ameliorated alveolar bone resorption. ADO = adenosine, AMP = adenosine monophosphate, ARs = adenosine receptors, CD25 = alpha‐chain from the interleukin‐2 receptor, CD73 = 5’‐ectonucleotidase, OBs = osteoblasts, OCs = osteoclasts, Pi = inorganic phosphate, RANK = receptor activator of nuclear factor κB, RANKL= RANK ligand, RATEVs = RATreg‐derived extracellular vesicles, RATregs = Retinoic acid‐induced Tregs, Teff = effector T cells, Tregs = regulatory T cells (Foxp3^+^ cells).

Accumulating evidence indicates that Treg‐derived EVs (TEVs) effectively mediate immunosuppression through their cargo enriched in regulatory factors, including CD73, positioning them as a promising therapeutic strategy for controlling inflammatory diseases (Rojas et al. 2020; Tung et al. 2020). In the present study, we demonstrated that Tregs induced with polarising cytokines in the presence of retinoic acid (RATregs) secrete CD73^+^ EVs, which generate adenosine upon AMP exposure. Importantly, these EVs suppressed the activation and proliferation of effector CD4^+^ T cells, in part through the action of the CD73/ADO pathway. Additionally, RATEVs modulated IL‐17A^+^ and RANKL^+^ CD4^+^ T cell‐mediated periodontal immune dysregulation, inhibited osteoclastogenesis and mitigated tooth‐supporting alveolar bone loss during experimental periodontitis (Figure 7).

Under inflammatory conditions and tissue damage, large amounts of ATP are released into the extracellular space, acting as a danger signal that perpetuates dysregulated inflammation (Kelestemur et al. 2023). The sequential hydrolysis of extracellular ATP by CD39 and CD73 into AMP and subsequently ADO plays a critical role in controlling inflammation and protecting tissues from further damage (Kelestemur et al. 2023). ADO interaction with its high‐affinity receptor A_2A_R, abundantly expressed on CD4^+^ T cells, induces intracellular cAMP accumulation, thereby inhibiting TCR signalling and suppressing effector T cell activation and proliferation (Cheng et al. 2022). In the present study, we demonstrated that RATregs and their secreted RATEVs exhibit high CD73 expression and are capable of hydrolysing AMP into ADO and inorganic phosphate. It is well established that TGF‐β can induce and sustain CD73 expression in activated murine CD4^+^ T cells (Regateiro et al. 2011). Moreover, stimulation with TGF‐β in combination with ATRA exerts a synergistic effect that significantly enhances CD73 expression, an effect not observed in natural Tregs under the same conditions (Mucida et al. 2009). Taken together, our observations complement these reports, supporting the finding of higher CD73 expression in iTregs than nTregs. Accordingly, CD73‐mediated ADO generation by RATEVs likely represents a key tissue‐protective mechanism, both by facilitating the clearance of pro‐inflammatory extracellular ATP and by promoting the suppression of effector CD4^+^ T cells, as demonstrated in our study.

The CD73/ADO pathway was the first immunosuppressive mechanism identified in nTreg‐derived EVs (Smyth et al. 2013). Along with IL‐35, Neuropilin‐1 (Nrp‐1) and specific microRNAs, CD73 is regarded as one of the key mediators of the immunoregulatory properties of murine TEVs (Campos‐Mora et al. 2022; Sullivan et al. 2020). Notably, the absence of CD73 on TEVs abrogates their immunosuppressive function (Smyth et al., 2013). Moreover, TEVs derived from Nrp‐1‐deficient Tregs exhibit reduced CD73 expression compared to TEVs from *wild‐type* Tregs and fail to suppress T cell activation and proliferation (Campos‐Mora et al. 2022). Beyond Tregs, CD73‐bearing EVs derived from B cells and CD8^+^ T cells have also been shown to promote immune suppression and control inflammation in a CD73‐dependent manner (Schneider et al. 2021; Zhang et al. 2019).

In this study, CD73‐mediated AMPase activity in RATEVs resulted in complete suppression of CD4^+^ T cells, but only in the presence of AMP. Thus, AMP exposure was essential for RATEV‐mediated immunosuppression, consistent with previous findings in CD73‐bearing EVs derived from human CD8^+^ T cells (Schneider et al. 2021). When CD4^+^CD73^−^ T cells were exposed to AMP in combination with a CD73 inhibitor, but without RATEVs, only limited suppression was observed; however, the presence of RATEVs significantly enhanced CD4^+^CD73^−^ T cell suppression. Interestingly, no suppression occurred when CD4^+^CD73^+^ T cells were exposed to AMP and CD73 inhibition without RATEVs. In contrast, when RATEVs were present, these cells were almost completely suppressed, despite CD73 blockade. These results suggest that when AMP is available and both RATEVs and target cells express CD73, the resulting environment becomes highly enriched in ADO, to a level that overcomes the effect of CD73 inhibition, leading to potent T‐cell suppression. As demonstrated in our experimental periodontitis model, this phenomenon could have therapeutic relevance: an ADO‐enriched microenvironment induced by RATEV administration may constrain inflammation by suppressing pro‐inflammatory T‐cell responses, thereby contributing to disease control. Moreover, it is plausible that Tregs, CD4^+^ T cells and/or CD8^+^ T cells infiltrating periodontal tissues may acquire CD73 expression in an environment saturated with CD73‐bearing RATEVs, an immunological process known as infectious tolerance, previously described for Treg‐derived EVs in other settings (Sullivan et al. 2020).

Increased evidence demonstrates that TEVs play essential regulatory roles across various pathological conditions. Dysregulation in the number or function of TEVs has been implicated in the pathogenesis of multiple diseases, including transplant rejection, autoimmune disorders, cancer and inflammatory conditions (Rojas et al. 2020). In this context, several studies have explored the immunoregulatory and therapeutic potential of TEVs. For instance, murine TEVs have been shown to ameliorate acute myocardial infarction by promoting macrophage M2 polarisation (Hu et al. 2020) and to suppress both peripheral and central nervous system inflammation in an LPS‐induced systemic inflammation model and a SOD1 mouse model of amyotrophic lateral sclerosis (Thome et al. 2022). Moreover, murine and human Treg‐derived EVs have been reported to prolong allograft survival by inhibiting T‐cell proliferation and pro‐inflammatory cytokine production (Tung et al. 2020). More recently, TEVs loaded with miR‐449a‐5p have been shown to restore the Th17/Treg balance and control rheumatoid arthritis (Chen et al. 2021). Notably, periodontitis shares pathogenic mechanisms with rheumatoid arthritis, characterised by Th17 cell expansion and impaired Treg function (Alvarez et al. 2019). Periodontitis, a chronic inflammatory disease driven by dysbiotic shifts in the subgingival microbiota, results in a disrupted Th17/Treg balance and subsequent tooth‐supporting alveolar bone loss (Hajishengallis and Lamont, 2021). Recent studies have highlighted the critical role of EVs in periodontitis pathogenesis (Cai et al. 2023). Indeed, EVs derived from periodontal bacteria act as carriers of virulence factors (Shi et al. 2020), whilst EVs produced by periodontal tissues and infiltrating immune cells can elicit either pro‐inflammatory or anti‐inflammatory responses (Han et al. 2022). Importantly, these host‐derived EVs can be detected in gingival crevicular fluid and saliva, offering potential diagnostic and prognostic biomarkers for periodontal disease (Han et al. 2022).

In recent years, EVs from various extra‐periodontal sources have been explored as potential therapeutic agents for periodontitis, yielding promising results (Cai et al. 2023). Using a ligature‐induced periodontitis model—such as the one employed in this study—periodontal inoculation of mesenchymal stem cell (MSC)‐derived EVs has been shown to promote M2 macrophage polarisation, thereby reducing inflammation and alveolar bone loss (Nakao et al. 2021). In the same experimental model, bone marrow‐derived MSC‐EVs enriched with miR‐1246 suppressed Th17 cell polarisation and restored Th17/Treg balance through the miR‐1246/Nfat5 and miR‐1246/ACE2/p‐YAP1 signalling axes (Xia et al. 2023). Additionally, MSC‐derived EVs have been proposed to enhance periodontal regeneration (Inchingolo et al. 2024).

In this study, treatment of experimental periodontitis with CD73‐enriched RATEVs markedly reduced the activation of the entire periodontal CD4^+^ T cell compartment. Notably, RATEV administration decreased the frequency of IL‐17A‐ and RANKL‐expressing CD4^+^ T lymphocytes. Given the well‐established role of IL‐17A and RANKL in driving inflammation and bone resorption in osteolytic diseases, including periodontitis (Alvarez et al. 2019), reducing these effector subsets supports the potent immunosuppressive activity of RATEVs. Interestingly, Tregs also exhibited a decreased frequency following RATEV treatment. In this context, it has been widely reported that CD4^+^Foxp3^+^ Tregs expand during periodontitis—and other dysbiosis‐driven inflammatory conditions—as a compensatory mechanism to counteract immune dysregulation (Alvarez et al. 2020; Tsukasaki et al. 2018). However, Tregs may become phenotypically unstable and functionally impaired under sustained inflammatory conditions, contributing to tissue destruction rather than resolution (Alvarez et al. 2020; Tsukasaki et al. 2018). In our model, RATEVs were administered on Days 3 and 6, corresponding to the early and peak phases of adaptive immune activation in the periodontium. We hypothesise that this timing fostered a suppressive environment that limited the inflammatory expansion or destabilisation of CD4^+^Foxp3^+^ Tregs. This may explain the reduction in Th17 lymphocytes and Tregs and the attenuation of alveolar bone loss. Supporting this, our data show that CD4^+^Foxp3^+^IL‐17A^+^ T lymphocytes—an unstable, osteoclastogenic Treg subset—were also significantly reduced after RATEV treatment. Additionally, we observed increased CD73 expression in effector CD4^+^ T lymphocytes and Tregs from cervical lymph nodes following RATEV administration. These findings suggest that these cells may have acquired CD73 from RATEVs and, potentially, immunosuppressive capacity; however, this possibility requires further investigation. Moreover, the concurrent acquisition of CD73 and decreased IL‐17A production by Tregs may reflect a partial restoration of their suppressive phenotype, which is often compromised under inflammatory stress (Alvarez et al. 2020). Lastly, the suppression of IL‐17A‐producing CD4^+^ T lymphocytes likely curtailed their osteoclastogenic activity, thereby contributing to bone preservation. In summary, CD73‐bearing RATEVs restored the periodontal Th17/Treg balance and suppressed osteoclastogenesis, ultimately mitigating alveolar bone loss.

During periodontitis, infected cells, periodontal pathogens and tissue injury promote the release of ATP into the periodontal environment (Yang et al. 2024). Extracellular ATP subsequently interacts with P2X_7_ purinergic receptors on fibroblasts and osteoblasts, triggering NLRP3 inflammasome activation and the production of pro‐inflammatory cytokines, thereby amplifying inflammation and promoting leukocyte recruitment (Binderman et al. 2007). Additionally, ATP‐P2X_7_ engagement induces the expression of RANKL, directly stimulating osteoclastogenesis and enhancing bone resorption (Binderman et al. 2007). Sequential hydrolysis of ATP and AMP by CD39 and CD73 generates abundant levels of ADO, with potent immunoregulatory properties. Indeed, the ADO‐A_2A_R interaction on T cells inhibits their activation, proliferation and pro‐inflammatory cytokine production (Allard et al. 2012). Moreover, ADO‐A_2A_R interaction in monocytes suppresses RANKL‐mediated osteoclast differentiation and function (Cheng et al. 2022). Thus, a dual mechanism could explain the therapeutic efficacy of CD73‐bearing RATEVs in controlling periodontitis. On the one hand, CD73‐expressing RATEVs may facilitate the removal of pro‐inflammatory extracellular ATP through its sequential hydrolysis into AMP and subsequently ADO. On the other hand, local ADO enrichment may inhibit A_2A_R‐expressing effector T cells and osteoclast precursors, ultimately restraining inflammation and bone loss.

Based on our findings, we propose that CD73 present in RATEVs sustains and amplifies ADO production as long as AMP is available in the local environment. However, CD73 expression patterns differ across species, and therefore, caution is warranted when extrapolating our results to human settings. Notably, Schneider et al. (2021) recently demonstrated that in humans, the stepwise hydrolysis of eATP is mediated by the coordinated action of CD39^+^ Treg‐derived EVs and CD73^+^CD8^+^ T cell‐derived EVs. These observations suggest that, to replicate the effects observed in our murine model, a combination of CD39^+^ Treg‐derived and CD73^+^CD8^+^ T cell‐derived EVs might be required to achieve efficient eATP degradation and ADO generation in humans. Nevertheless, further characterisation of both EV subtypes is necessary to validate this hypothesis. Importantly, CD73‐mediated ADO production may not be the sole mechanism underlying the immunomodulatory effects of RATEVs in periodontitis. One limitation of our study is the lack of comprehensive profiling of the RATEV cargo. Therefore, we cannot exclude that other immunoregulatory components, such as miRNAs, cytokines, or enzymes, may contribute to their suppressive effects. Indeed, several miRNAs carried by murine TEVs, including Let‐7b, Let‐7d and miR‐155, have been shown to suppress effector T cell activation and pro‐inflammatory cytokine secretion (Torri et al., 2017). Similarly, human TEVs display a distinct miRNA signature, with enrichment of miR‐146a‐5p, miR‐150‐5p and miR‐21‐5p, and depletion of miR‐106a‐5p, miR‐155‐5p and miR‐19a‐3p (Tung et al. 2020). Functionally, TEVs—but not Th1/Th17‐derived EVs—exert potent suppressive effects on CD4^+^ T cells (Torri et al. 2017). In addition, EV‐mediated immunosuppression has been attributed to other factors, such as the inducible nitric oxide synthase (iNOS) enzyme. For example, iNOS carried by dnIKK2‐Treg‐derived EVs can be transferred to naïve T cells, where it inhibits cell cycle progression and induces apoptosis (Tung et al. 2020). Similarly, TEVs carrying the IL‐35 subunits Ebi3 and p35 can transfer these proteins to T and B cells, enabling them to acquire immunosuppressive properties (Sullivan et al. 2020). Therefore, besides the CD73/ADO pathway explored in this study, other regulatory molecules present in RATEVs may contribute to CD4^+^ T cell suppression and the control of periodontitis.

In this study, RATEVs were used instead of RATregs to treat periodontitis, primarily due to the potential phenotypic instability of RATregs in inflammatory environments such as the periodontitis‐affected tissues. Under these conditions, Tregs may lose Foxp3 expression and transdifferentiate into IL‐17A‐ and RANKL‐producing exFoxp3 Th17‐like cells, thereby exacerbating disease progression (Alvarez et al. 2020; Tsukasaki et al. 2018). In contrast, RATEVs provide a stable and predictable immunomodulatory effect, as their molecular cargo is not susceptible to inflammatory reprogramming (Rojas et al. 2020). To optimise treatment efficacy and target critical phases of periodontitis progression, RATEVs were administered at two strategic time points. Early inoculation on Day 3 was intended to limit innate immune cell infiltration, whilst administration on Day 6 aimed to modulate adaptive immune activation and osteoclastogenesis, thereby maximising the therapeutic potential of RATEVs in controlling immune dysregulation and alveolar bone loss.

Similar to RATEVs, EVs derived from other cellular sources, such as dental pulp stem cells (DPSCs) and periodontal MSCs, have shown therapeutic potential for treating periodontitis (Inchingolo et al. 2024). However, the composition of EV cargo can vary markedly depending on the tissue origin and the inflammatory status of the donor, which may result in inconsistent therapeutic outcomes (Gao et al. 2016). In contrast, RATEVs are produced by Tregs, cells with inherent immunosuppressive properties, and their therapeutic efficacy is further potentiated by RA stimulation, which enhances EV production and optimises cargo content. Moreover, CD73 activity in RATEVs promotes sustained ADO generation, conferring dual benefits in terms of immunosuppression and bone preservation (Kelestemur et al. 2023). These results are consistent with recent studies reporting that bone loss can be ameliorated through CD39‐ and CD73‐mediated ATP hydrolysis driven by T cell‐derived apoptotic EVs (Yang et al. 2025). Although direct ADO administration could be considered an alternative strategy, its clinical utility is limited due to rapid extracellular metabolism and clearance. Specifically, ADO is quickly converted into inosine by adenosine deaminase (ADA) or is taken up by cells via nucleoside transporters such as equilibrative nucleoside transporters (ENTs), which are key regulators of extracellular ADO levels (Pastor‐Anglada and Pérez‐Torras 2018). RATEVs, by contrast, may overcome these limitations by continuously generating ADO in situ via CD73, sustaining their immunoregulatory and bone‐protective effects over time. Compared to conventional periodontal therapies, RATEVs represent an innovative approach that targets host immune dysregulation rather than focusing exclusively on bacterial control. Although mechanical debridement and antibiotic therapy effectively reduce microbial burden, they do not address the underlying immune imbalance and may contribute to antimicrobial resistance or systemic side effects. In contrast, RATEVs modulate the periodontal immune response and preserve alveolar bone integrity, positioning them as a promising therapeutic strategy for periodontitis. Nevertheless, challenges such as large‐scale production, targeted delivery methods and the assessment of long‐term safety must be addressed prior to their translation into clinical practice.

Although this study provides robust evidence supporting the role of CD73/ADO signalling in mediating the immunosuppressive and anti‐osteolytic effects of RATEVs on CD4^+^ T lymphocytes and alveolar bone loss in experimental periodontitis, several key questions remain unanswered. In particular, the precise mechanisms by which RATEVs exert their effects remain to be elucidated. Future studies should investigate the specific pathways involved in attenuating inflammation, the upregulation of CD73 expression in T cells, and the potential transfer of CD73 to other immune cells infiltrating periodontal tissues or cervical lymph nodes. In this sense, TEVs can interact with surface receptors on target cells or via internalisation, enabling the delivery of their cargo and subsequent modulation of cellular functions (Chen et al. 2021; Rojas et al. 2020; Tung et al. 2020). However, in this study, the exact mechanism of action of RATEVs was not established. Additional experiments using TEVs derived from T cells not exposed to RA or exposing RATEVs to CD73‐deficient T cells obtained from CD73 knock‐out mice would be valuable to clarify the specific contribution of CD73/ADO signalling to the observed outcomes. Moreover, the effects of ADO are context‐dependent and influenced by the repertoire of GPCRs expressed in target cells. In particular, A_2A_R‐expressing T lymphocytes are highly responsive to ADO‐mediated immunosuppression (Gourdin et al. 2018). Therefore, analysing the GPCR expression profile in IL‐17A‐ and RANKL‐producing CD4^+^ T lymphocytes, as well as IL‐17A‐producing Tregs infiltrating the periodontium of RATEV‐treated animals, would help determine whether the observed effects are indeed dependent on the CD73/ADO axis.

Another important consideration is the functional stability of RATEVs within infected periodontal tissues. Pathogenic bacteria associated with human periodontitis produce high levels of proteolytic enzymes, which may impair immune cell function and potentially degrade the immunosuppressive cargo carried by RATEVs (Tubero Euzebio Alves et al. 2024). Although widely used, the ligature‐induced periodontitis model differs from human disease in several critical aspects, including the composition of the dysbiotic biofilm and the extent and nature of the inflammatory response. These differences may influence the stability and efficacy of RATEVs in vivo, thereby limiting the direct translational applicability of our findings to human periodontitis. In addition, evaluating the long‐term therapeutic efficacy of RATEVs and their safety profile in chronic inflammatory settings is essential to support their clinical development. Future studies should assess potential adverse effects and determine whether repeated or sustained RATEV administration maintains efficacy without inducing tolerance or off‐target effects. Addressing these knowledge gaps will provide a more comprehensive understanding of RATEV functionality and inform their potential as a therapeutic tool for treating inflammatory diseases such as periodontitis.

This study proposes a novel and promising therapeutic strategy based on the local administration of CD73‐enriched RATEVs to deal with periodontitis. RATEVs offer several unique advantages: (1) TEVs are produced by Tregs, per excellence suppressor cells, constituting a key component of their suppressive arsenal. (2) Tregs generated in the presence of RA, a potent inducer of CD73, produce RATEVs highly enriched in CD73. (3) RATEVs exert immunosuppressive effects independently of their parent cells, offering a cell‐free alternative to Treg‐based therapies. (4) Unlike Tregs, which may lose their suppressive phenotype under inflammatory conditions such as periodontitis, RATEVs retain their stability and immunosuppressive function. (5) CD73 confers dual protective effects by promoting the degradation of pro‐inflammatory extracellular ATP and generating ADO, a potent immunosuppressant with additional anti‐osteolytic properties. (6) RATEVs may also transfer CD73 to other immune cells, including CD4^+^ and CD8^+^ T cells and dysfunctional Tregs, potentially restoring their regulatory capacity. In summary, our findings demonstrate that RATEVs suppress CD4^+^ T‐cell‐mediated inflammatory and osteolytic responses and ameliorate alveolar bone loss in experimental periodontitis, likely through mechanisms involving the CD73/ADO signalling pathway.

## Author Contributions

**Claudia Terraza‐Aguirre**: investigation (supporting); methodology (supporting). **Lesley A Smyth**: conceptualization (lead); data curation (equal); formal analysis (equal); funding acquisition (equal); investigation (equal); methodology (equal); resources (equal). **Carolina Rojas**: conceptualization (lead); data curation (lead); formal analysis (lead); investigation (lead); methodology (lead); project administration (lead); visualization (lead); writing–original draft (lead); writing–review and editing (lead). **Michelle García**: investigation (supporting); methodology (supporting); project administration (supporting). **Luis González‐Osuna**: investigation (supporting); methodology (supporting); writing–review and editing (supporting). **Mauricio Campos‐Mora**: formal analysis (supporting); investigation (supporting); methodology (supporting); validation (supporting). **Enrique Ponce de León**: investigation (supporting); methodology (supporting). **Alfredo Sierra‐Cristancho**: investigation (supporting); methodology (supporting); writing–review and editing (supporting). **Cristian Cortez**: methodology (supporting). **Luis Daniel Sansores‐España**: investigation (supporting); methodology (supporting); writing–review and editing (supporting). **Paola Carvajal**: conceptualization (supporting); project administration (supporting). **Jordan Bazoer**: investigation (supporting); methodology (supporting). **Qi Peng**: formal analysis (equal); investigation (equal); methodology (equal). **Charlotte Lawson**: investigation (supporting); methodology (supporting). **Karina Pino‐Lagos**: conceptualization (equal); data curation (equal); funding acquisition (equal); investigation (lead); resources (equal); supervision (equal); writing–original draft (equal); writing–review and editing (equal). **Rolando Vernal**: conceptualization (equal); data curation (equal); formal analysis (equal); funding acquisition (lead); project administration (equal); resources (equal); supervision (equal); writing–review and editing (equal).

## Conflicts of Interest

The authors declare no conflicts of interest.

## Supporting information




**Supplementary Figure 1. RATreg characterization. (a)** Flow cytometry gating strategy used to analyze the purity of CD4^+^ T cells obtained from the spleen of C57BL/6 Foxp3^GFP+^ mice. The sequential gating strategy was based on FSC‐A/SSC‐A parameters, FSC‐A/FSC‐H singlet discrimination, and live/dead cell staining. **(b)** Flow cytometry gating strategy used to analyze the Foxp3, CD25, and CD73 expression in induced RATregs. **(c)** Comparison of CD73 expression among natural Tregs (nTregs), Tregs induced with polarizing cytokines without RA (iTregs), and RATregs analyzed by flow cytometry. Histograms show comparative CD73 MFI. The consecutive graph shows CD73 MFI quantification among RATregs, iTregs, and nTregs (*p<0.05). **(d)** Topographic expression of CD4 and CD25 in CD4^+^ T cells before (upper panels) and after (lower panels) their induction towards RATregs, analyzed by imaging flow cytometry.

**Supplementary Figure 2. Western blot membranes. (a)** Full Western blot membrane image showing CD73, β‐actin, and CD81 detection. A picture of the original membrane is included to indicate the sections where the membrane was cut to prevent secondary antibody cross‐reactivity. **(b)** Full Western blot membrane image for Calnexin, β‐actin, and CD9 detection. A picture of the original membrane is included to indicate the sections where the membrane was cut to prevent secondary antibody cross‐reactivity.

**Supplementary Figure 3**. **RATEVs titration for suppression assays,** **sorting parameters** **for** **C** **D4** ^**+**^ **CD73** ^**−**^ **and CD4** ^**+**^ **CD73** ^**+**^ **T‐cell purification, and CD73 expression in CD4** ^**+**^ **CD73** ^**−**^ **and CD4** ^**+**^ **CD73** ^**+**^ **T cells in response to RATEV treatment**. Erythrocyte‐depleted splenocytes labelled with Cell Trace VioletTM (CTV) were activated *in vitro* with soluble anti‐CD3ε (1 µg/mL) and treated with RATEVs (1, 2.5 and 5 × 10^8^ particles, termed RATEVs‐lo, RATEVs‐hi, and RATEVs‐very hi, respectively) for 72 h. Non‐activated (NA) or activated (Act) untreated cells (exposed to the vehicle) were defined as negative and positive activation controls, respectively. **(a)** Representative histograms that show CD4^+^ T cell proliferation (CTV dilution) in the following conditions: Non‐activated (gray line), untreated activated (red line), and RATEVs‐treated activated (light and dark blue lines) cells. Each histogram peak represents a proliferation cycle in which parent cells are divided. The graph shows the proliferation index values as mean±SD (*p<0.05; **p<0.01; ****p<0.0001; ns = non‐significant). **(b)** Representative dot plots and quantification graphs of the mean percentage for activated (CD25^+^) CD4^+^ T cells. **(c)** Cell sorting gating strategy used to obtain the responder CD4^+^CD73^−^ and CD4^+^CD73^+^ T cells. Cells were isolated from C57BL/6 Foxp3^GFP+^ transgenic mice by selecting them according to the FSC‐A/SSC‐A parameters, FSC‐A/FSC‐H singlet discrimination, live/dead cell staining, and the CD4 (positive expression), Foxp3 (negative expression), and CD73 (positive or negative expression) markers. Two cell populations were selected from the CD4^+^Foxp3^−^ T cells: CD73^+^ (upper gating box) and CD73^−^ (lower gating box) cells. **(d)** Analysis of the purity of the obtained CD4^+^CD73^−^ and CD4^+^CD73^+^ responder T cells. **(e)** CD4^+^CD73^−^ or (f) CD4^+^CD73^+^ sorted responder T cells were activated with anti‐CD3ε (5 µg/mL) and anti‐CD28 (µg/mL) in the presence of EHNA (10 µM) and NBTI (10 µM) and then incubated with or without 5’‐AMP (50 µM), RATEVs (1 or 2.5 × 10^8^ particles, termed RATEVs‐lo and RATEVs‐hi, respectively), and/or two CD73 inhibitors (ADPan or APCP, 100 µM). T‐cell CD73 expression levels were assessed by flow cytometry.

**Supplementary Figure 4. Biodistribution and permanence at the inoculation site of DiR‐labeled RATEVs. (a)** Near‐infrared fluorescence shows DiR‐labelled RATEVs biodistribution at the following evaluation time points: baseline, 30 min, 24 h, 48 h, 72 h, and 7 days. **(b)** In addition, analysis was performed directly in the maxilla, liver, and kidney organs on day 7. On the close‐up images of the maxilla (upper box), the red dashed line circle shows the puncture site where DIR‐stained RATEVs or control solution was inoculated.

**Supplementary Figure 5. Gating strategy of cells obtained from periodontal palatal mucosa and cervical lymph nodes. (a)** Periodontal mucosa and **(b)** cervical lymph node cells were selected as single, live, CD45^+^, and CD4^+^/CD8^+^ T cells. On the CD4^+^ T cell population, Tregs and conventional T (convT) cells were defined according to their Foxp3 expression. **(c)** Unstained control taken as reference for CD73 expression determination on cervical lymph node‐derived T cells.

## Data Availability

The data that support the findings of this study are available from the corresponding author upon reasonable request.
