# Supplementary figures and images for: Induced Treg‐Derived Extracellular Vesicles Suppress CD4+ T‐Cell‐Mediated Inflammation and Ameliorate Bone Loss During Periodontitis Partly Through CD73/Adenosine‐Dependent Immunomodulatory Mechanisms
Source: J Extracell Vesicles. 2025 Jul 7;14(7):e70118. doi: 10.1002/jev2.70118 (PMC12230360; doi:10.1002/jev2.70118)

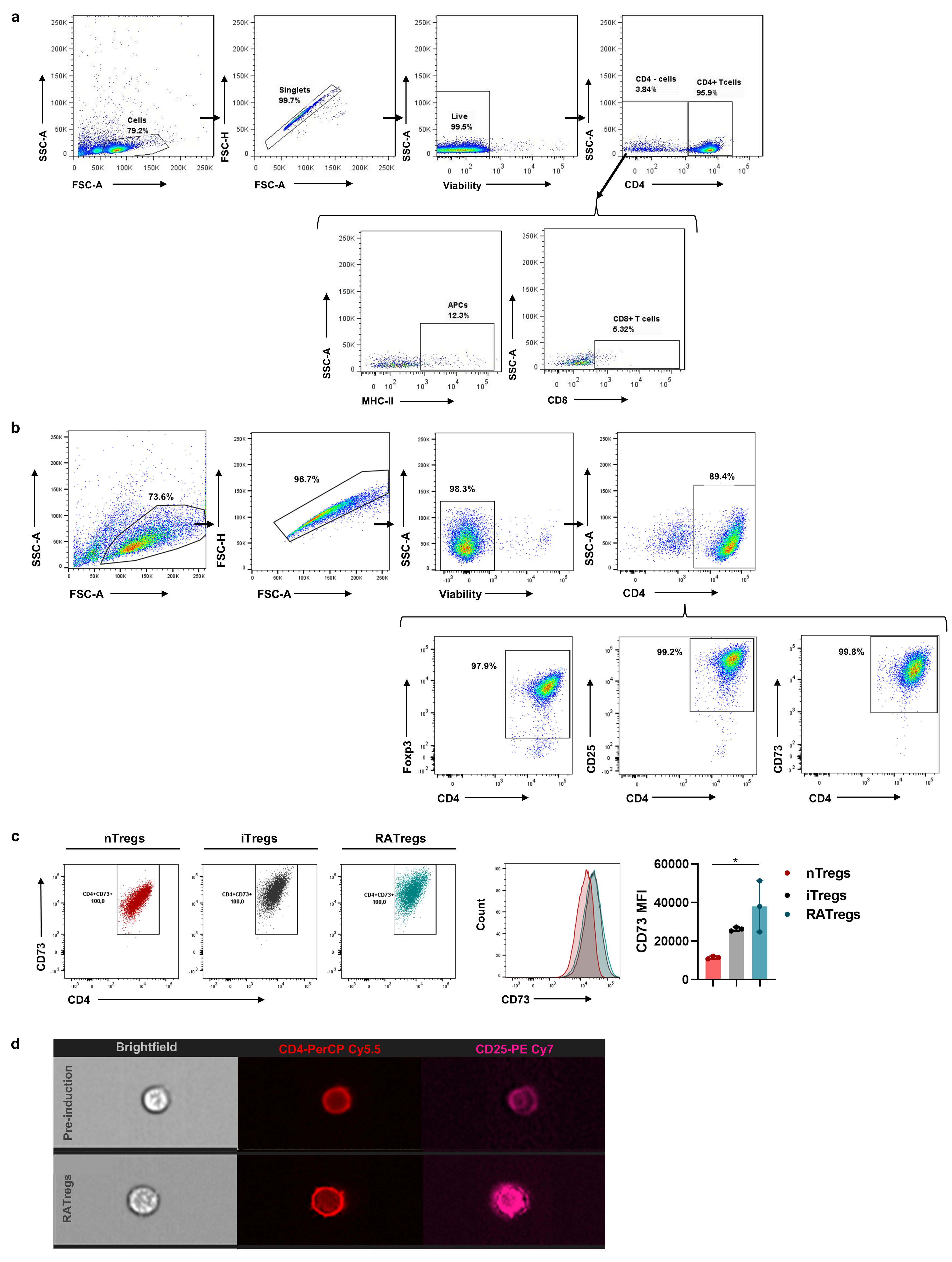

Supplement: Supplementary file 1 — Supplementary Figure 1. RATreg characterization. (a) Flow cytometry gating strategy used to analyze the purity of CD4+ T cells obtained from the spleen of C57BL/6 Foxp3GFP+ mice. The sequential gating strategy was based on FSC‐A/SSC‐A parameters, FSC‐A/FSC‐H singlet discrimination, and live/dead cell staining. (b) Flow cytometry gating strategy used to analyze the Foxp3, CD25, and CD73 expression in induced RATregs. (c) Comparison of CD73 expression among natural Tregs (nTregs), Tregs induced with polarizing cytokines without RA (iTregs), and RATregs analyzed by flow cytometry. Histograms show comparative CD73 MFI. The consecutive graph shows CD73 MFI quantification among RATregs, iTregs, and nTregs (*p<0.05). (d) Topographic expression of CD4 and CD25 in CD4+ T cells before (upper panels) and after (lower panels) their induction towards RATregs, analyzed by imaging flow cytometry. [file JEV2-14-e70118-s004.TIF]

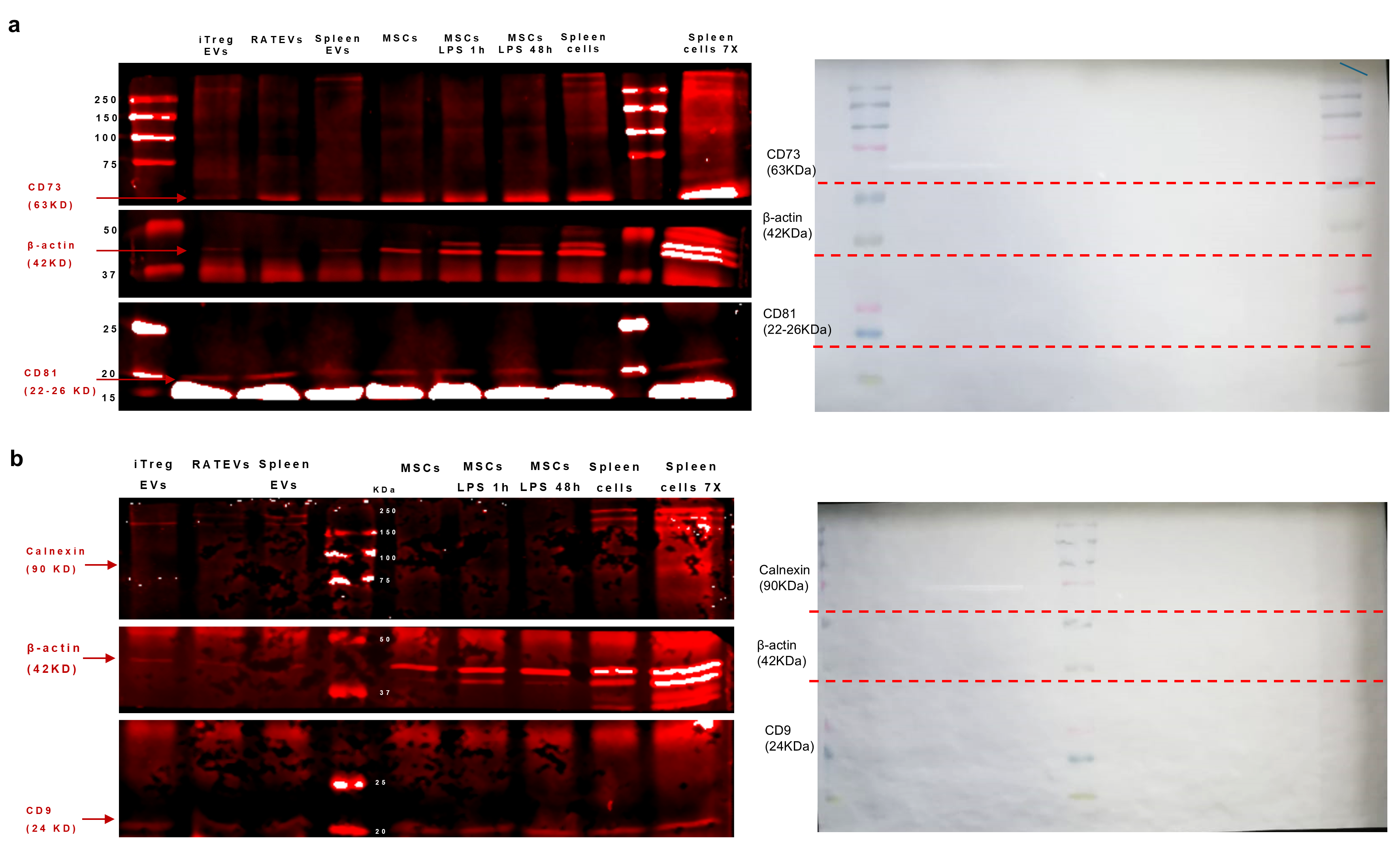

Supplement: Supplementary file 2 — Supplementary Figure 2. Western blot membranes. (a) Full Western blot membrane image showing CD73, β‐actin, and CD81 detection. A picture of the original membrane is included to indicate the sections where the membrane was cut to prevent secondary antibody cross‐reactivity. (b) Full Western blot membrane image for Calnexin, β‐actin, and CD9 detection. A picture of the original membrane is included to indicate the sections where the membrane was cut to prevent secondary antibody cross‐reactivity. [file JEV2-14-e70118-s003.TIF]

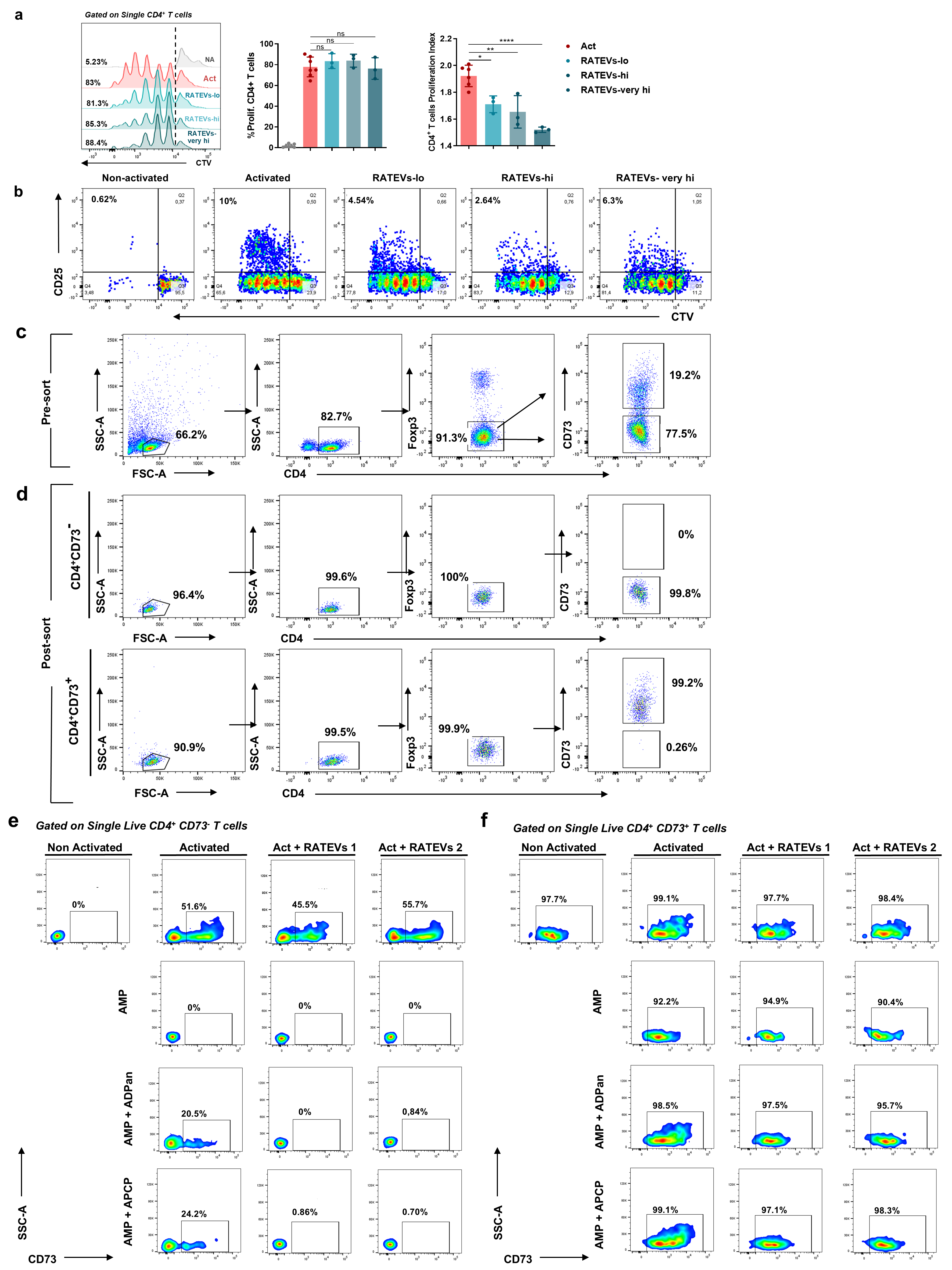

Supplement: Supplementary file 3 — Supplementary Figure 3. RATEVs titration for suppression assays, sorting parameters for C D4 + CD73 − and CD4 + CD73 + T‐cell purification, and CD73 expression in CD4 + CD73 − and CD4 + CD73 + T cells in response to RATEV treatment. Erythrocyte‐depleted splenocytes labelled with Cell Trace VioletTM (CTV) were activated in vitro with soluble anti‐CD3ε (1 µg/mL) and treated with RATEVs (1, 2.5 and 5 × 10^8^ particles, termed RATEVs‐lo, RATEVs‐hi, and RATEVs‐very hi, respectively) for 72 h. Non‐activated (NA) or activated (Act) untreated cells (exposed to the vehicle) were defined as negative and positive activation controls, respectively. (a) Representative histograms that show CD4+ T cell proliferation (CTV dilution) in the following conditions: Non‐activated (gray line), untreated activated (red line), and RATEVs‐treated activated (light and dark blue lines) cells. Each histogram peak represents a proliferation cycle in which parent cells are divided. The graph shows the proliferation index values as mean±SD (*p<0.05; **p<0.01; ****p<0.0001; ns = non‐significant). (b) Representative dot plots and quantification graphs of the mean percentage for activated (CD25+) CD4+ T cells. (c) Cell sorting gating strategy used to obtain the responder CD4+CD73− and CD4+CD73+ T cells. Cells were isolated from C57BL/6 Foxp3GFP+ transgenic mice by selecting them according to the FSC‐A/SSC‐A parameters, FSC‐A/FSC‐H singlet discrimination, live/dead cell staining, and the CD4 (positive expression), Foxp3 (negative expression), and CD73 (positive or negative expression) markers. Two cell populations were selected from the CD4+Foxp3− T cells: CD73+ (upper gating box) and CD73− (lower gating box) cells. (d) Analysis of the purity of the obtained CD4+CD73− and CD4+CD73+ responder T cells. (e) CD4+CD73− or (f) CD4+CD73+ sorted responder T cells were activated with anti‐CD3ε (5 µg/mL) and anti‐CD28 (µg/mL) in the presence of EHNA (10 µM) and NBTI (10 µM) and then incubated with or without 5’‐A [file JEV2-14-e70118-s001.TIF]

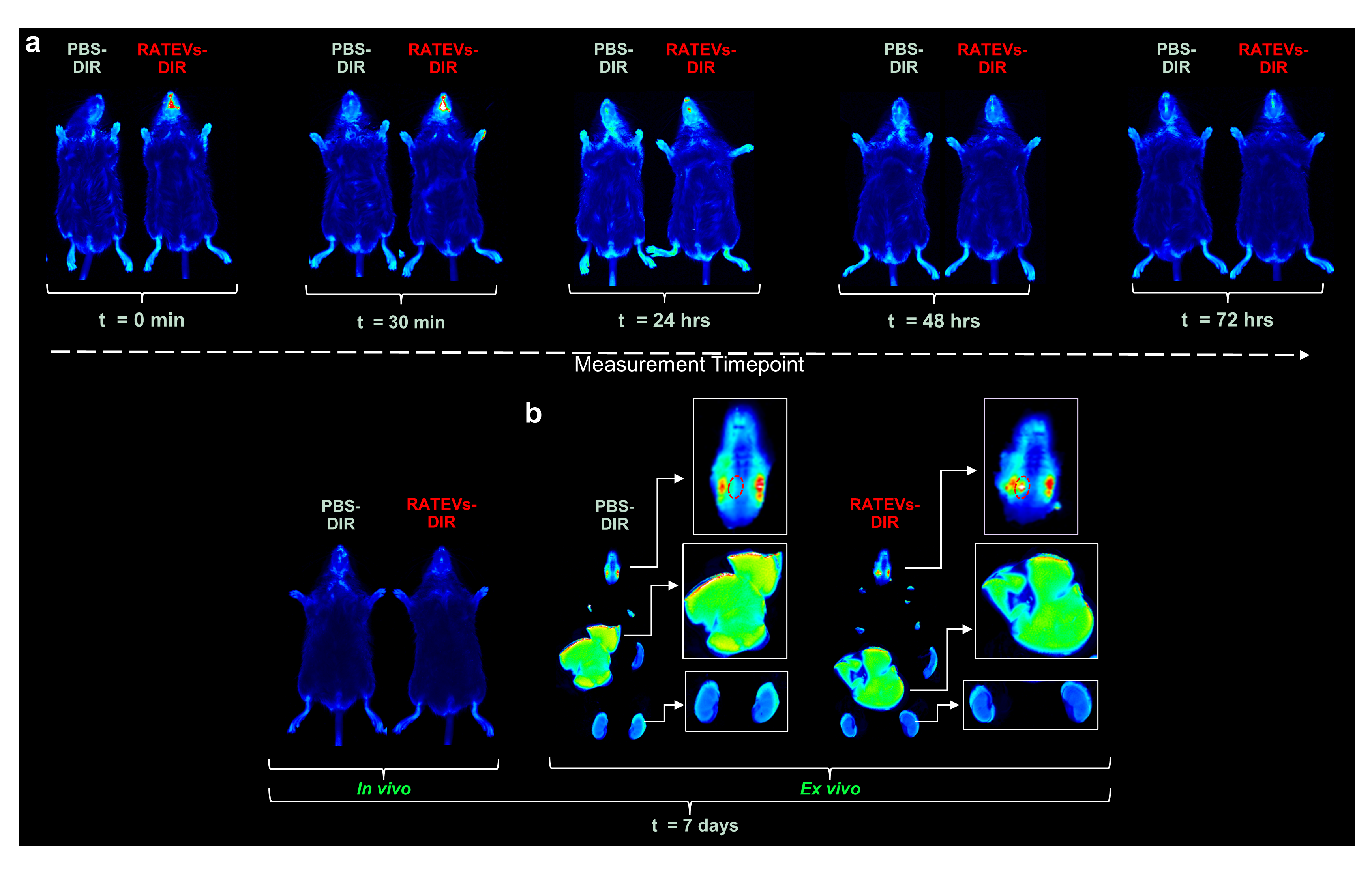

Supplement: Supplementary file 4 — Supplementary Figure 4. Biodistribution and permanence at the inoculation site of DiR‐labeled RATEVs. (a) Near‐infrared fluorescence shows DiR‐labelled RATEVs biodistribution at the following evaluation time points: baseline, 30 min, 24 h, 48 h, 72 h, and 7 days. (b) In addition, analysis was performed directly in the maxilla, liver, and kidney organs on day 7. On the close‐up images of the maxilla (upper box), the red dashed line circle shows the puncture site where DIR‐stained RATEVs or control solution was inoculated. [file JEV2-14-e70118-s002.TIF]

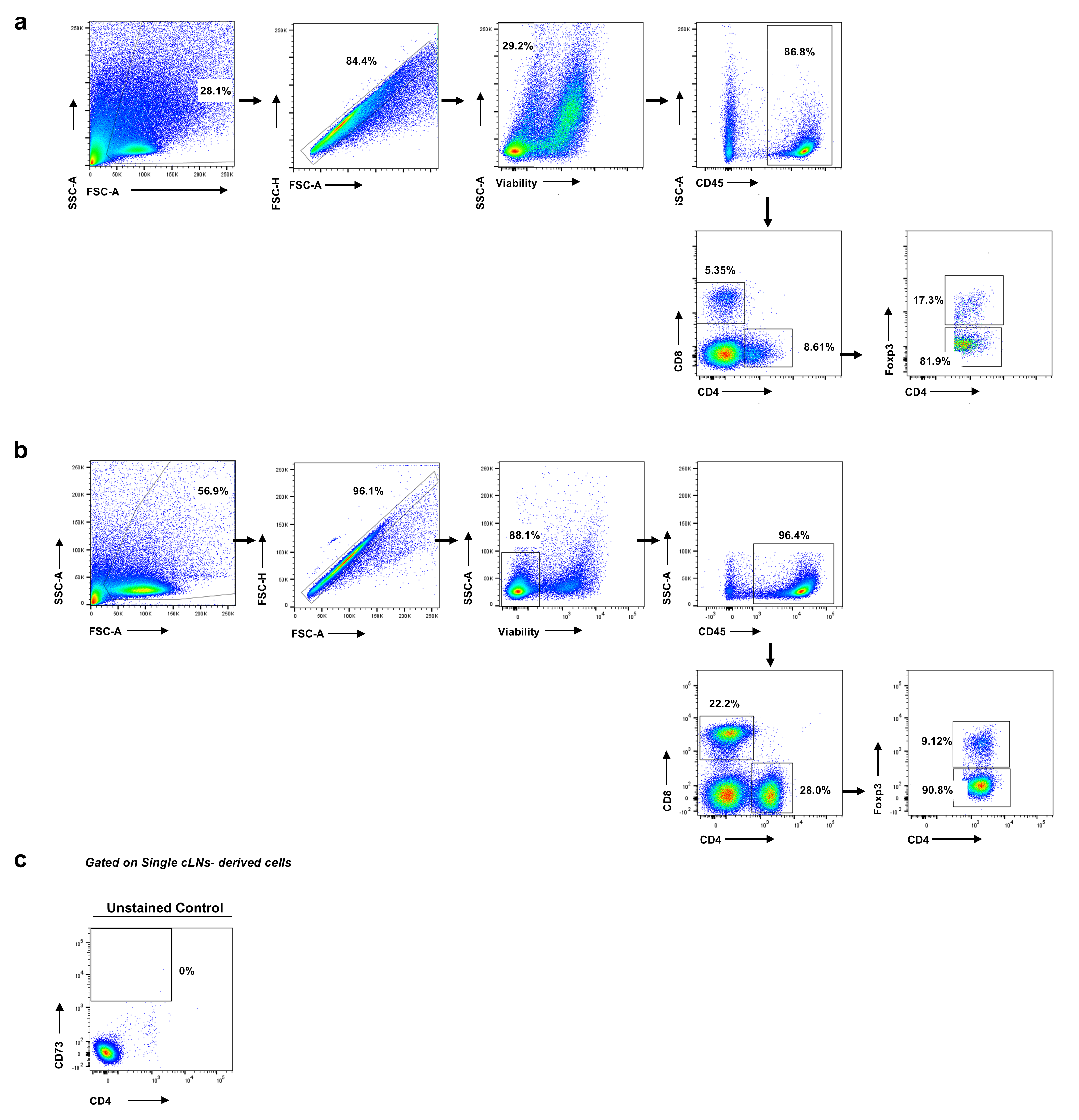

Supplement: Supplementary file 5 — Supplementary Figure 5. Gating strategy of cells obtained from periodontal palatal mucosa and cervical lymph nodes. (a) Periodontal mucosa and (b) cervical lymph node cells were selected as single, live, CD45+, and CD4+/CD8+ T cells. On the CD4+ T cell population, Tregs and conventional T (convT) cells were defined according to their Foxp3 expression. (c) Unstained control taken as reference for CD73 expression determination on cervical lymph node‐derived T cells. [file JEV2-14-e70118-s005.TIF]
